# Engineering an antimicrobial chimeric endolysin that targets the phytopathogen *Pseudomonas syringae* pv. *actinidiae*

**DOI:** 10.1016/j.jbc.2025.110224

**Published:** 2025-05-09

**Authors:** Suzanne L. Warring, Hazel M. Sisson, George Randall, Dennis Grimon, Dorien Dams, Diana Gutiérrez, Matthias Fellner, Robert D. Fagerlund, Yves Briers, Simon A. Jackson, Peter C. Fineran

**Affiliations:** 1 Department of Microbiology and Immunology, University of Otago, Dunedin, New Zealand; 2 Maurice Wilkins Centre for Molecular Biodiscovery, University of Otago, Dunedin, New Zealand; 3 Genetics Otago, University of Otago, Dunedin, New Zealand; 4 Bioprotection Aotearoa, University of Otago, Dunedin, New Zealand; 5 Department of Biochemistry, University of Otago, Dunedin, New Zealand; 6 Department of Biotechnology, Ghent University, Ghent, Belgium

**Keywords:** endolysin, bacteriophages, *Pseudomonas syringae*, protein engineering, biocontrol

## Abstract

Global food shortages and rising antimicrobial resistance require alternatives to antibiotics and agrichemicals for the management of agricultural bacterial pathogens. The phytopathogen *Pseudomonas syringae* pv. *actinidiae* (*Psa*) is the causal agent of kiwifruit canker and is responsible for major agricultural losses. Bacteriophage enzymes present an emerging antimicrobial option. Endolysins possess the ability to cleave peptidoglycan and are effective antimicrobials against gram-positive bacteria. Delivery of endolysins to the peptidoglycan of gram-negatives is impeded by the additional outer membrane. To overcome this barrier, we used VersaTile molecular shuffling to produce *Psa*-targeting chimeric proteins which were then tested for antimicrobial activity. These chimeras consist of endolysins linked by polypeptides to diverse phage proteins mined from *Psa* phage genomes. A preferential configuration for antibacterial activity was observed for enzymatic domains at the N terminus and alternative phage proteins at the C terminus. The lead variant possessed an N-terminal modular endolysin and a C-terminal lipase. Antibacterial activity was enhanced with the addition of the chemical permeabilizers such as citric acid or EDTA. Mutagenesis of the lipase active site eliminated exogenous antibacterial activity toward *Psa*. The endolysin-lipase chimera demonstrated specificity toward *Psa*, illustrating potential as a targeted biocontrol agent. Overall, we generated a chimeric endolysin with exogenous and specific activity toward *Psa*, the causative agent of kiwifruit canker.

Bacterial pathogens that cause significant impacts in agriculture are displaying increased resistance to current antimicrobial and agrichemical treatments (1, 2). Therefore, there is a need for alternative approaches. *Pseudomonas syringae* pv. *actinidiae* (*Psa*) (3) is a phytopathogen of kiwifruit, with biovar 3 responsible for the pandemic which emerged in 2008 and has remained an ongoing challenge to global kiwifruit production (4). Management of *Psa* involves replacing cultivars with more tolerant varieties and orchard management practices that include copper and antibiotic sprays (5). Increasing observations of agrichemical resistance, particularly to copper (6, 7), coupled with mandates to remove traditional antibiotics from food production, has led to research groups examining bacteriophages as potential biocontrol agents for *Psa* (8, 9, 10, 11, 12, 13).

Another approach is to utilize antimicrobial enzymes from phages known as endolysins (14). Endolysins are produced at the end of the lytic lifecycle, released across the inner membrane of bacteria by holins to then enzymatically degrade peptidoglycan, causing cell lysis and release of phage progeny (15). Exogenous application of endolysins successfully lyses gram-positive bacteria due to their external peptidoglycan, but lysis of gram-negatives is impeded due to the impermeable outer membrane (16). The outer membrane of gram-negatives can be overcome by fusion of endolysins with outer-membrane-permeabilizing peptides (OMPs), trademarked by Lysando as Artilysins (17). Although OMPs can enhance endolysin access across the outer membrane, they are often inactivated in various contexts, such as human serum, which reduces their efficacy (18). OMP fused endolysins can possess species-specific antimicrobial activity but are often active against a range of gram-negatives (16). Nevertheless, they remain more targeted than broad-spectrum antibiotics. An alternative and pathogen-specific approach has been demonstrated by the C terminal fusion of a T5-phage receptor-binding protein (RBP) to an endolysin to produce an “Innolysin” with exogenous antibacterial activity against *Escherichia coli* (19). Innolysins have also been engineered against *Campylobacter jejuni* (20).

Here, we expanded these concepts by fusing endolysins (enzymatically active domains; EADs) to a broad selection of phage proteins, including holins, tail fibers, RBPs and virion-associated lysins (VALs), bioinformatically mined from a *Psa* phage collection (8, 9, 10, 21). By using VersaTile DNA shuffling (22), we produced chimeric phage protein fusion libraries and identified a lead candidate, containing a lipase and an endolysin, which exhibited moderate exogenous antimicrobial activity against *Psa*. This chimera showed specific activity against *Psa* even in synergy with a permeabilizing organic acid. Overall, we have generated a potential control agent having antimicrobial properties against the gram-negative kiwifruit pathogen *Psa.*

## Results

### VersaTile production of chimeric endolysin libraries with activity against *Psa*

To generate chimeric phage-derived antimicrobial enzymes against *Psa*, it was first necessary to identify proteins of interest to include in our combinatorial libraries. We used eight diverse phage genomes across three different morphotypes (Table S1) to find proteins for inclusion in our repository. Previous work demonstrated that phage RBPs could be fused to endolysins to produce chimeric endolysins with exogenous antibacterial activity against specific gram-negative bacteria (19). Therefore, we posited that additional phage proteins such as tail fibers, baseplate proteins, tail spikes, holins, tail sheath proteins, and VALs could also enhance antibacterial activity of endolysins. Each of these classes of phage proteins has the potential to either bind or lyse bacteria and represent an untapped potential in endolysin engineering (23). To maximize diversity in the repository, proteins selected had <90% amino acid sequence identity to homologous proteins from our *Psa* phage subset. The initial repository included five endolysins (EADs), three holins, five baseplate associated proteins, two tail spike proteins, 21 tail fiber and receptor binding domains (RBPs) and 14 VALs. For larger proteins, phage parts were delineated, which involved cloning an individual domain from the full-length coding sequence using domain boundary prediction tools to preserve the secondary structure of the selected domain (24). The seven polypeptide linkers used in this work were previously developed (20). All of the linkers used in this work, except for linker 8, are rigid coils/helices of varying length derived from viral sources (25). Linker 8 is a flexible linker derived from Lysostaphin (26). The amino acid sequence of EADs, polypeptide linkers, and phage parts in the final repository are provided in Table S2.

**Figure 1:**
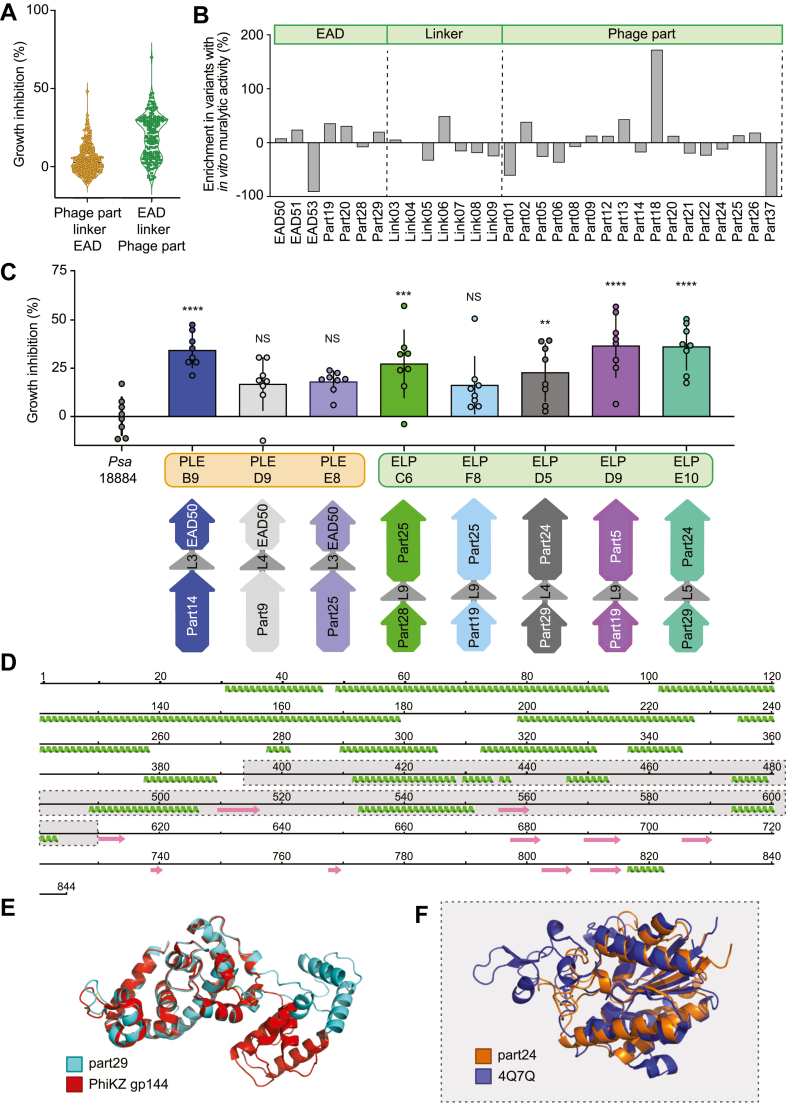
**Medium-throughput screening of chimeric *Psa* phage endolysin libraries identifies variants that show ∼50% growth inhibition of *Psa*.***A*, growth inhibition (%) of *Psa* in response to endolysin phage-protein fusion variants (*A*_600_ relative to the *A*_600_ of a negative control well (GFP lysate added)). Two configurations of variant libraries, phage-part–linker–EAD (PLE) and EAD–linker–phage-part (ELP), were tested. Representative raw *A*_600_ data, from which growth inhibition was calculated, is shown in Fig. S1, *A* and *B*. *B*, enrichment of EADs, linkers, and parts in muralytically active variants relative to total variant libraries of the ELP configuration; determined by Nanopore sequencing. *C*, replicate growth inhibition data for lead variants from initial screens with composition and configuration shown below. Statistical analysis was performed using one-way ANOVA analysis relative to the GFP lysate control added to *Psa* WT (∗∗∗∗*p* < 0.0001. ∗∗∗*p* < 0.0005, ∗∗*p* < 0.01). Data are presented as the mean ± SD, each data point represents a biological replicate. *D*, secondary structure prediction by Phyre2 of full amino acid sequence from which part24 was delineated, with extracted region marked by *gray box*. *Green coils* indicate alpha helices and *pink arrows* indicate beta strands. *E*, 3D superposition of a ColabFold structural prediction of part29 (*cyan*) with the crystal structure of gp144 from PhiKZ 3BKH (*red*) (30). RMSD value of 0.8 Å. *F*, 3D superposition of a ColabFold structural prediction of part24 (*orange*) with the crystal structure of a putative lipase from *Chitinophaga pinensis* DSM 2588 4Q7Q (*purple*). RMSD value of 1.7 Å. RMSD values were determined at the C-α position. EAD, enzymatically active domain.

To produce variant libraries for activity screening from our repository of *Psa* phage proteins, we used VersaTile DNA shuffling (22). Variant libraries were produced with EAD and phage parts (P) connected by short polypeptide linkers in two configurations: phage-part–linker–EAD (PLE) and EAD–linker–phage-part (ELP). Across the two libraries, the theoretical complexity accounted for ∼2200 chimeric endolysins. For our initial medium-throughput screening of antimicrobial activity of variant libraries against *Psa*, we selected 236 and 173 random isolates from the PLE and ELP configurations, respectively. These were grown and expressed and crude lysates from variant library expressions were screened for growth inhibition against *Psa* WT. The average growth inhibition of the PLE configuration was ∼3%, while for the ELP configuration it was ∼19% (Fig. 1*A*). Such data provided initial evidence that the ELP configuration had a higher antibacterial activity.

To determine which phage parts produced active variants, we used a high-throughput (HT) *in vitro* assay to screen the ELP configuration for peptidoglycan degradation. This HT method allows thousands of variants to be screened in a single assay with muralytic activity indicated by the formation of a halo around variant *Escherichia coli* pLysS colonies. To determine which parts were enriched in active variants, DNA from both the total and active variant libraries was sequenced with long-read technology and compared (Fig. 1*B* and Table S3). Several parts were enriched in the active subset, including EAD parts 19 and 20 (jumbo phage SLT-transglycosylase domains), linker 6, and phage parts 2 (ΦPsa315 baseplate wedge), 13 (delineated RBP from a T7-like tail fiber), and 18 (jumbo phage virion structural protein). Parts with decreased abundance included EAD53, part1 (*Psa* myovirus holin), part6 (a delineated PAAR domain from a predicted jumbo phage tail fiber) and part37 (siphovirus tip attachment protein). Purification of EAD53 (Fig. S1*C*) showed poor expression of the endolysin, which may account for the underrepresentation of these parts in the active library.

To further validate the medium and HT approaches, we identified the composition of eight lead variants from Figure 1*A* (>30% growth inhibition) and replicated their growth inhibition using the medium-throughput approach (Fig. 1*C* and Table S4). Three variants showed >36% inhibition (PLE-B9, ELP-D9, and ELP-E10) and were therefore carried forward for further testing. Variant PLE-B9 contained a delineated lyase from ΦPsa17 at the N terminus and an endolysin from ΦPsa374 at the C terminus (EAD50). The variant ELP-D9 possessed part19 at the N terminus and part5 (a PAAR motif containing protein (27)) at the C terminus. ELP-E10 had part29 at the N terminus (an endolysin from a jumbo phage) and part24 at the C terminus (a delineated lipase domain from a siphovirus belonging to the *Nickievirus* genus). By comparing the enriched parts in the HT screen to the medium-throughput growth inhibition assays, we identified part19, part25, and part29 as favorable. Interestingly, HT-enriched parts 2, 13, and 18 and linker 6 were absent in lead variants from growth inhibition assays. Across both screens, EAD53, part1, part6, and part37 were absent in active variants, indicating they should be excluded in subsequent assemblies. The differences between HT peptidoglycan degradation and antimicrobial assays exemplifies that lower throughput growth inhibition assays are preferable for identifying exogenously active endolysin fusions.

### The lead antimicrobial candidate contains an endolysin fused to a putative lipase

To assess the efficacy of lead candidates, PLE-B9, ELP-D9, and ELP-E10 were purified and tested in muralytic and antibacterial assays. The candidate PLE-B9 displayed no exogenous effect in antibacterial assays (Fig. S1, *D* and *E*) and therefore was not pursued further. The combination of candidates with EDTA was also assessed because chelating agents synergize with endolysins to promote antibacterial activity against gram-negative bacteria (18, 28). ELP-E10 was prioritized as it exhibited enhanced growth inhibition against *Psa* when combined with 0.1 mM EDTA in IC_50_ assays compared with ELP-D9 (Fig. S2*B*). The endolysin domain of ELP-E10 (part29), is homologous to gp144 of PhiKZ, a *Pseudomonas aeruginosa* jumbo phage (29) (Figs. 1*E* and S3*A*). The gp144 endolysin is a highly lytic modular peptidoglycan hydrolase, with a peptidoglycan binding domain at the N terminus and a lytic transglycosylase at the C terminus (30, 31). The C-terminal domain of ELP-E10 is part24 which is a lipase domain derived from a phage with a siphovirus morphology. The gray box in Figure 1*D* shows the region of the protein that has been cloned to make part24 based on secondary structure predictions. In related phages, phiK7B1, ZY21, hairong, and nickie, the protein is annotated as a GDSL-like lipase or hydrolase (32). GDSL lipolytic enzymes are a distinct and divergent subclass of esterases and lipases that possess a GDSL amino acid sequence motif, and they often possess broad substrate specificity (33). The protein used to generate part24 is aligned to homologous proteins in Fig. S3*B*. In psageB1, this protein is annotated as a lysis associated protein (34). The predicted structure of part24 was compared with a similar lipase from *Chitinophaga pinensis* DSM 2588 and demonstrated the domain was appropriately delineated to preserve predicted secondary structure and enzymatic activity (Fig. 1*F*).

To further study the antibacterial and muralytic activity of ELP-E10, it was purified by nickel affinity chromatography (*via* C-terminal His_6_-tag) and size-exclusion chromatography (SEC) (Fig. S4). ELP-E10 eluted at ∼55 kDa, yielding ∼65 mg/L from 1 L of culture and was confirmed by mass spectrometry (Fig. S5). ELP-E10 rapidly degraded the peptidoglycan of *P. aeruginosa* PAO1 spheroplasts with a calculated specific activity of ∼339,000 U/mg (Fig. S6), which is comparable to the PhiKZ endolysin (210,000 U/mg) (31) and indicates that the endolysin retains a high level of lytic activity when fused to a lipase. In summary, we have generated, and purified to high yield, a lead antimicrobial chimera with a modular endolysin with high lytic activity and a C-terminal putative lipase.

### ELP-E10 is active against *Psa* and synergizes with permeabilizing chemicals

**Figure 2:**
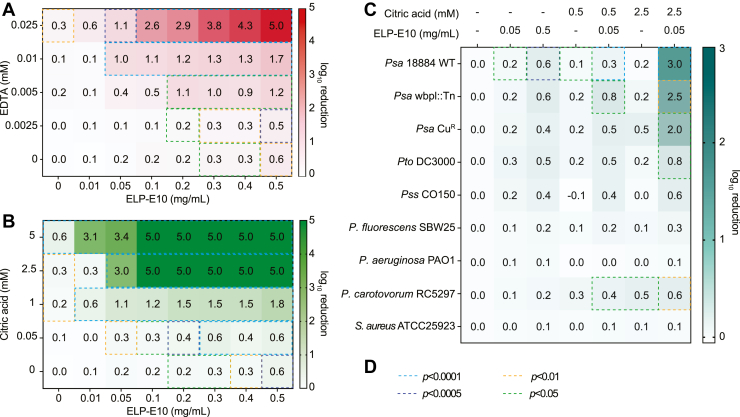
**The chimeric endolysin ELP-E10 has exogenous antibacterial activity against *Psa***. *A* and *B*, Checkerboard assays showing antibacterial activity, expressed as log_10_ reduction of CFU/ml, of *Psa* treated with varying concentrations of ELP-E10 and *A*, EDTA or *B*, citric acid. *C*, Log_10_ reduction of various bacteria treated with 0.05 or 0.5 mg/ml ELP-E10 and 0, 0.5, or 2.5 mM citric acid. *A*–*C* are presented as the mean log-fold change in CFU/ml. *Psa* Cu^R^ represents copper resistant *Psa* ICMP 246510. *D*, statistical analysis of *A*–*C* was performed on CFU/ml data (Fig. S7) using one-way ANOVA analysis relative to applicable control, with significant differences highlighted in *dashed boxes* in *A*–*C* as indicated. In Fig. S7, data are presented as the mean ± SD and each data point is a biological replicate. The limit of detection was 5-log reduction. CFU, colony-forming units; ELP, EAD–linker–phage-part.

To assess the antibacterial potential of ELP-E10 against *Psa*, the purified enzyme was tested in antibacterial assays with and without the permeabilizers, EDTA and citric acid. Chelating agents such as EDTA and citric acid permeabilize the outer membrane of gram-negative bacteria and enhance the lytic capacity of endolysins. First, we tested ELP-E10 without permeabilizers, which displayed moderate exogenous antibacterial activity against *Psa*, with 0.5 mg/ml resulting in a ∼0.6-log fold reduction (Fig. 2, *A*–*C*). In comparison, 0.2 mg/ml Innolysin Ec21 elicited a ∼2 to 3 log reduction in *E. coli* (19) and 1 mg/ml of Innolysin Cj1 reduced *C. jejuni* by ∼1 to 1.5 log (20). Notably, activity of both Innolysin variants in the absence of permeabilizers was improved through substitution of the endolysin, peptide linker or tail fiber component, providing engineering possibilities to improve exogenous antibacterial activity of the ELP-E10 parental chimera.

Next, the antimicrobial activity of ELP-E10 was assessed at a range of protein and EDTA concentrations (Fig. 2*A*). The addition of 0.01 mM EDTA enhanced the antimicrobial activity of 0.5 mg/ml ELP-E10 against *Psa*, with a 1.66-log fold reduction detected (Fig. 2*A*). Application of EDTA in the environment is undesirable due to its environmental persistence and effects on metal bioavailability in soils (35). Therefore, we assessed the synergy of ELP-E10 with an alternative environmentally sustainable chelating agent; citric acid (Fig. 2*B*). Citric acid permeabilizes the outer membrane of gram-negatives through chelation and acidic effects and is already used in kiwifruit orchards to remove water spots on fruit prior to harvest https://www.eastpack.co.nz/vdb/document/11314 (accessed 2024-06-17). We observed a >5-log reduction in *Psa* with 0.1 mg/ml ELP-E10 and 2.5 mM citric acid, compared with a 0.3-log decrease with 2.5 mM citric acid alone.

To evaluate the synergy between permeabilizers and ELP-E10, the fractional inhibitory concentration index (FICI) was calculated, with values ≤ 0.5 indicating synergy (36). The calculated FICIs for ELP-E10 with EDTA or citric acid were 0.27 and 0.22, respectively, indicating synergy of ELP-E10 with both chemical permeabilizers. Importantly, the observed synergy, which results in over 5-log reduction in *Psa*, utilizes concentrations of citric acid that are lower than already used in kiwifruit orchards, suggesting combinations of ELP-E10 with citric acid may be appropriate for biocontrol of *Psa*. In fact, our chimera may be an improved biocontrol compared to single phages alone. Indeed, we previously demonstrated *in planta* that a single phage against *Psa* only caused an initial 2-log reduction in bacterial density (21). In conclusion, the chimera has moderate exogenous antibacterial activity against *Psa* and demonstrates synergy when combined with either EDTA or citric acid.

### ELP-E10 exhibits a narrow antimicrobial spectrum toward *P. syringae* pathovars

To assess the antimicrobial spectrum of ELP-E10, we tested the chimera alone and in combination with citric acid against relevant pathogens and a commensal soil bacterium (37) (Fig. 2*C*). First, we hypothesized that *Psa* strains that were resistant to other treatments such as phages (*via* lipopolysaccharide (LPS) mutation) or copper would remain sensitive to the chimera. Indeed, an LPS-attenuated strain of *Psa* 18884 (*wbpl*::Tn) (38) and a copper resistant *Psa* isolate ICMP 246510 (39) were as susceptible to 0.5 mg/ml ELP-E10 as *Psa* 18884. In addition to its effects against *Psa* strains, ELP-E10 had exogenous activity when applied alone to *P. syringae* pv. *tomato* (*Pto*) DC3000 (40) and *P. syringae* pv. *syringae* (*Pss*) CO150 (41), which infect tomato and cherry plants, respectively. Next, we wanted to assess the susceptibility of different bacteria when exposed to both ELP-E10 and citric acid. We previously showed that treatment with the ΦPsa374 endolysin and 5 mM citric acid reduced the cell viability of all tested *Pseudomonas* pathovars below the limit of detection, hindering assessment of variance in activity for endolysin and citric acid combinations (42). Based on the previous data, we assessed the combination of ELP-E10 with lower concentrations of citric acid (0.5 and 2.5 mM) so that subtle differences in antimicrobial activity of ELP-E10 against different *Pseudomonas* pathovars could be more readily quantified. For most strains, 0.5 or 2.5 mM citric acid alone did not reduce their viability by > 0.3 log. However, the copper resistant *Psa* strain and a *Pectobacterium carotovorum* (43) strain showed 0.5-log fold reductions with 2.5 mM citric acid alone; indicating partial susceptibility to low concentrations of citric acid. Interestingly, the combination of 2.5 mM citric acid and ELP-E10 exclusively showed significant reductions of 2-3-log fold in cell viability for *Psa* strains, while *Pto* and *Pss* strains displayed lower susceptibility (0.8-log and 0.6-log reductions, respectively). Therefore, full synergistic effects of ELP-E10 used in concert with citric acid were limited to *Psa*. Importantly, a commensal soil microbe *Pseudomonas fluorescens* was not impacted by ELP-E10 alone or in combination with 2.5 mM citric acid. Similarly, the pathogens *P. aeruginosa* (44) and *Staphylococcus aureus* (45) were not susceptible, illustrating the targeted antimicrobial spectrum of the chimera. In summary, when used in conjunction with citric acid, the endolysin-lipase chimera is specific toward *Psa* and has limited activity toward a representative commensal.

### Chimeric configuration affects antibacterial activity

**Figure 3:**
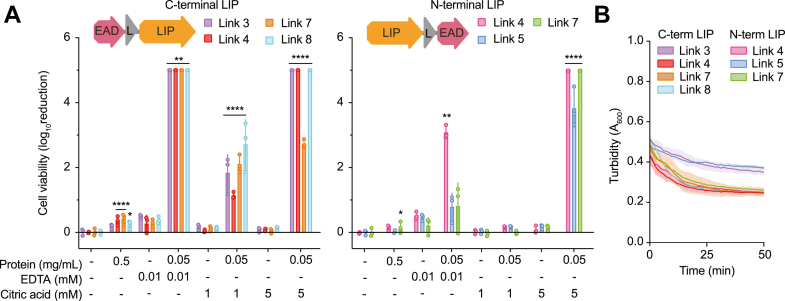
**Chimera configuration and polypeptide linker affect antibacterial activity and synergy with chemical permeabilizers**. *A*, log fold reduction of ELP-E10 variants with differing configurations and linkers in the presence of 1 or 5 mM citric acid or 0.01 mM EDTA against *Psa*. Limit of detection: 5-log. Statistical analysis was performed using one-way ANOVA analysis relative to applicable control from CFU/ml data (Fig. S8). ∗∗∗∗*p* < 0.0001, ∗∗*p* < 0.01, ∗*p* < 0.05. Data are presented as the mean ± SD, and each data point is a biological replicate. *B*, muralytic assays of 0.5 μg/ml ELP-E10 configuration variants. Data are presented as the mean ± SEM of technical triplicates. CFU, colony-forming units; ELP, EAD–linker–phage-part.

To determine if configuration and peptide linker affected antibacterial activity of the chimeric endolysin, variants were produced with the lipase at either the N or C terminus of the protein with different peptide linkers. Antibacterial activity of the variants against *Psa* was tested in the absence or presence of EDTA or citric acid (Fig. 3*A*). Endolysin fusions showed greater antibacterial activity with the EAD at the N terminus and the lipase domain (LIP) at the C terminus. Indeed, treatment of *Psa* with the C-terminal lipase variants resulted in no colonies with the addition of 0.01 mM EDTA or 2-3-log fold killing with 1 mM citric acid (Fig. 3*A*). In contrast, the N-terminal lipase variant only had one variant (linker 4) that exhibited significant killing with 0.01 mM EDTA. Such data are consistent with our original library screen which showed that higher growth inhibition with phage parts at the C-terminal end of endolysin fusions (Fig. 1*A*). Additionally, increased synergy with the addition of 1 mM citric acid was observed for peptide linkers 3, 7, and 8 when compared with the parental ELP-E10 (Fig. 2*B*), indicating that peptide linkers can enhance antibacterial activity. However, no clear trends emerged from the data to provide insight into linker properties such as the length or rigidity that resulted in the observed increased synergy. Rigid helical polypeptide linkers are thought to more efficiently allow domains to retain their individual functionality, while flexible linkers often result in poor expression or loss of activity of domains (25, 46). The chimeras with different configurations of the EAD and lipase domains retained muralytic activity but EAD-link3-LIP and LIP-link5-EAD had reduced activity (Fig. 3*B*). Notably, the parental ELP-E10 possesses linker 5 and is highly muralytically active (Fig. S6). Therefore, linker 3 in ELP and linker 5 in PLE configurations may have a minor effect on enzymatic activity of the fusion endolysins. Importantly, for variants with linkers 4 and 7, but with EAD and lipase domains in opposite configurations, there was no difference in muralytic activity. Therefore, the differences in antibacterial activity in Figure 3*A* are likely due to the positioning of the lipase domain rather than the EAD. In summary, configuration of the *Psa* endolysin-lipase chimeras does not affect muralytic activity, but a C-terminal lipase domain results in increased antibacterial activity and linker substitution can increase the synergy of the chimera with citric acid.

### Separate endolysin and lipase domains of ELP-E10 are ineffective as exogenous antimicrobials

**Figure 4:**
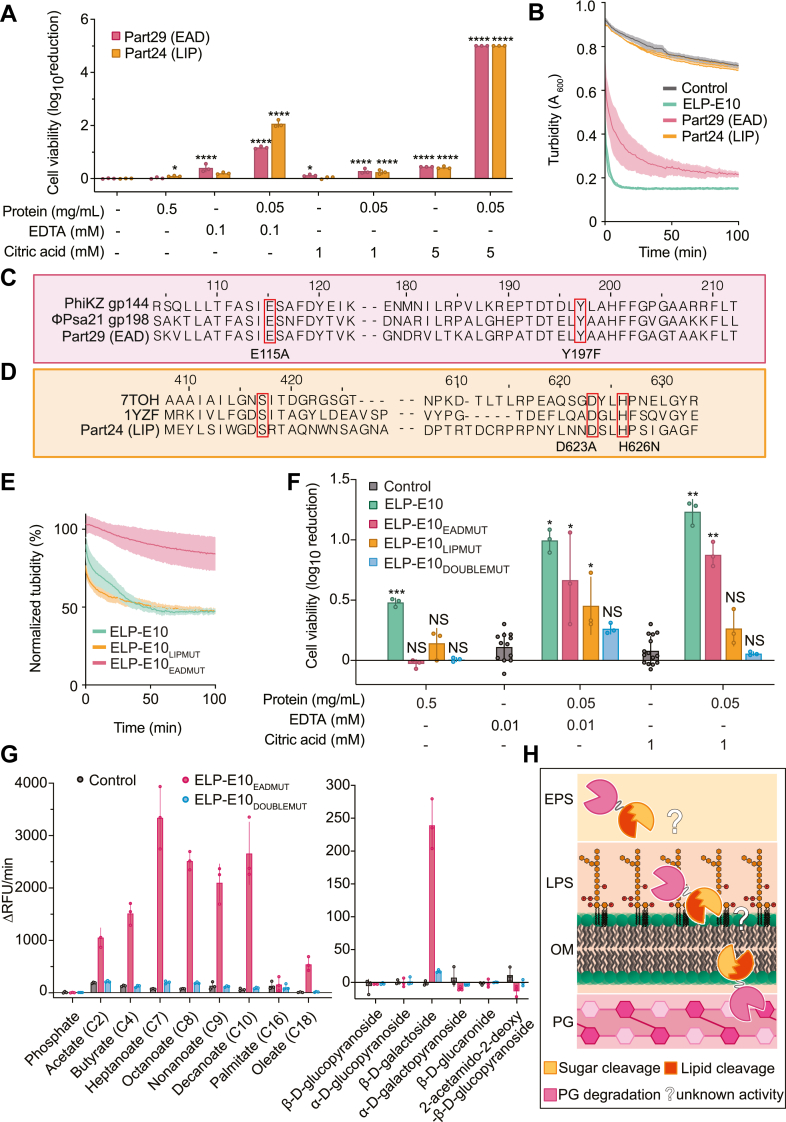
**Actives sites of lipase and endolysin domains contribute to the exogenous antimicrobial activity of ELP-E10.***A*, antibacterial activity of ELP-E10 individual domains against *Psa* at different protein, citric acid, and EDTA concentrations. *B*, muralytic assays of 0.5 μg/ml part24, part29, and ELP-E10. *C* and *D*, identification of residues predicted to be responsible for (*C*) muralytic activity of part29 and (*D*) lipase activity of part24. *E*, muralytic activity of ELP-E10 and mutants, normalized to the no protein control. *F*, antibacterial assay against *Psa* WT of ELP-E10 and mutants alone and combined with chemical permeabilizers. Muralytic activity of ELP-E10 and mutants, normalized to the no protein control. *G*, esterase activity of ELP-E10_EADMUT_ and ELP-E10_DOUBLEMUT_ against 4-MU model substrates with lipid chains of various length and sugars (the full data set is provided in Fig. S9*A*). Data are presented as the mean ± SD, individual data points represent technical triplicates. Biological replicate data are presented in Fig. S9. *H*, proposed model for exogenous antibacterial action of ELP-E10. In *A* and *F* data are presented as the mean ± SD and data points are biological replicates. Statistical analysis was performed using one-way ANOVA analysis relative to applicable control from CFU/ml data (Fig. S8). ∗∗∗∗*p* < 0.0001, ∗∗∗*p* < 0.0005, ∗∗*p* < 0.01, ∗*p* < 0.05. In *B* and *E* data are presented as the mean ± SEM of technical triplicates. 4-MU, 4-methylumbelliferyl; CFU, colony-forming units; ELP, EAD–linker–phage-part.

To test if exogenous activity of ELP-E10 requires both endolysin and lipase domains rather than individual domains, each protein was individually purified and confirmed by mass spectrometry (Fig. S5) and tested in antibacterial and muralytic assays (Fig. 4, *A* and *B*). Seventy milligrams of the endolysin domain (part29), and ∼0.04 mg for the lipase domain (part24) were obtained from 200 ml of culture following immobilized nickel affinity chromatography purification. These yields imply that the endolysin has greater soluble expression than the lipase and that their fusion improves lipase expression and purification compared to individual expression of the lipase domain. Both domains were examined separately for antibacterial and muralytic activity (Fig. 4, *A* and *B*). There was no significant exogenous antibacterial activity from the endolysin domain against *Psa*. However, the lipase domain demonstrated very minor antibacterial activity (0.06-log fold, *p* = 0.03) (Fig. 4*A*). Importantly, the antibacterial activity of the chimera ELP-E10 was ∼10-fold more than the lipase alone (Fig. 2). When combined with 0.01 mM EDTA, the lipase exhibited the strongest synergy with enhanced antibacterial activity (∼2-log fold) compared with the endolysin alone, or the chimera (both ∼1-log fold). The increased killing capacity of the lipase domain when used in conjunction with EDTA, coupled with the minor exogenous activity of the lipase alone illustrates that this domain possesses a degree of antibacterial activity. Importantly, when compared to the separate domains, the chimera displayed the highest antibacterial activity when combined with 1 mM citric acid. Furthermore, 5 mM citric acid combined with 50 μg/ml of either the endolysin or lipase domain alone resulted in >5-log fold killing of *Psa*, with no colonies detected. As expected, no muralytic activity was detected for the lipase, as seen for other phage lipases (47), while the endolysin domain had strong muralytic activity (Fig. 4*B*). In conclusion, fusion of the lipase to the endolysin increases the yield and antimicrobial synergy with citric acid. Overall, the chimera is more effective than individual domains as an antimicrobial against *Psa*.

### Lipase and EAD active sites are required for exogenous antibacterial activity

To determine if enzymatic function of each domain within the chimera was essential for its exogenous antibacterial activity, point mutations were introduced to abolish the muralytic function of the endolysin and serine hydrolase activity of the lipase (Fig. 4, *C* and *D*). To identify mutations that disrupt the endolysin activity of part29, we examined a homolog (gp144) from the *Pseudomonas* jumbo phage PhiKZ. In gp144, muralytic activity was abolished with E115A and Y197F mutations, so we introduced equivalent mutations to the chimera (Fig. 4*C*, red boxes) (48). In agreement, mutation of these residues in the EAD of ELP-E10 (named ELP-E10_EADMUT_) abolished muralytic activity of the chimera (Fig. 4*E*). Additionally, exogenous antibacterial activity of ELP-E10_EADMUT_ was decreased significantly, and synergy with 1 mM citric acid reduced moderately (Fig. 4*F*). To inactivate the lipase, we identified and mutated aspartate and histidine residues (ELP-E10_LIPMUT_; shown in boxes in Fig. 4*D*) that were previously demonstrated to remove serine hydrolase activity of a homologous lipase in *Aeromonas hydrophila* (49). Indeed, mutations at these conserved residues in the ELP-E10 lipase abolished the exogenous antibacterial effect against *Psa* (Fig. 4*F*). Furthermore, ELP-E10_LIPMUT_ had decreased synergy with 1 mM citric acid compared with the parental chimera. Mutation of catalytic sites in both domains (ELP-E10_DOUBLEMUT_) yielded a chimera that caused no significant reduction of *Psa*, irrespective of the presence of permeabilizers. In summary, catalytic sites in both the endolysin and lipase domains are required for optimal exogenous activity of the chimera.

To identify the esterase activity and ensure the lipase (part24) was enzymatically active in the endolysin fusion, we utilized a 4-methylumbelliferone (4-MU) fluorogenic assay with varying length lipid chains and various sugar groups. To ensure any observed activity was due to the lipase domain we used ELP-E10_EADMUT_, which had no muralytic activity from the endolysin domain (Fig. 4*E*). ELP-E10_EADMUT_ showed activity toward lipid chains of C2–C10 length (Fig. 4*G*). Interestingly, 4-MU β-D-galactoside was cleaved but not any of the other sugar substrates that were tested (Fig. 4*G*). While this assay has previously demonstrated lipase/esterase activity toward lipids for bacterial lipases (50, 51, 52) and cleavage of 4MU-galactose by galactosidases (53, 54, 55), a lipase with activity toward both lipids and sugars has not previously been demonstrated (56). Additionally, the ELP-E10_DOUBLEMUT_ did not cleave any substrates tested and was comparable to the no enzyme control (Fig. 4*G*), providing further evidence that the activity seen with the ELP-E10_EADMUT_ was due to the lipase activity. Lipases are uncommon phage depolymerases with only a few identified bioinformatically in *Cellulophaga* and *Pseudomonas* phages (57). In *Bacillus* phage ΦNIT1, the lipase PghP was not directly associated with virions but was released upon bacterial lysis for breakdown of extracellular polymeric substance (EPS) that would ordinarily impede phage infection in stationary phase (47). In the *Psa* phage genome, the gene from which part24 is derived is upstream of a holin and endolysin, likely forming a lysis cassette for the phage (Fig. S9*B*). An AlphaFold3 model of the full protein shows that it includes a helix, the lipase/hydrolase domain and a carbohydrate-binding domain (CBD) (Fig. S9, *B*–*D*). The CBD is structurally analogous to a CBD at the C terminus of a modular *Clostridium thermocellum Ct*Cel9D-Cel44A cellulase implicated in plant cell wall degradation (58), whereby the CBD binds 1,4-glucans. Multiple lines of evidence lead us to propose that the lipase within ELP-E10 breaks down an unknown substrate on the surface *Psa* such as EPS or LPS, allowing the endolysin access to peptidoglycan; (1) the activity of the lipase toward 4MU-galactose and lipids, (2) the genetic context in a lysis cassette within phage genome and (3) the function of lipases in other phages, such as PghP, that breakdown EPS. A schematic in Figure 4*H* shows our proposed model for the mechanism of exogenous antibacterial activity of ELP-E10. In summary, we have demonstrated that the lipase domain retains esterase activity when fused to an endolysin and is essential for exogenous antibacterial activity of ELP-E10.

## Discussion

We have engineered and characterized a chimeric protein (ELP-E10), which has exogenous antibacterial activity against the gram-negative kiwifruit pathogen *Psa*. The chimera has a modular endolysin from a *Psa* jumbo phage at the N terminus, a peptide linker and a lipase from a *Psa* siphovirus at the C terminus. The endolysin is similar to the endolysin gp144 from *P. aeruginosa* phage PhiKZ (29), and the lipase domain is similar to a lipase from *C*. *pinensis* DSM 2588. ELP-E10 reduced the viable count of WT *Psa* (biovar 3, ICMP 18884) by ∼0.6-log without the addition of chemical permeabilizers. Additionally, ELP-E10 has a narrow antibacterial spectrum that includes *P. syringae* pathovars, illustrating its suitability for environmental application with permeabilizers such as citric acid for biocontrol of plant pathogens. Furthermore, the lipase must be at the C terminus for full antibacterial activity of the chimera and different linkers can enhance the antimicrobial synergy with citric acid. Finally, we illustrated that the exogenous antibacterial activity of ELP-E10 required catalytic activity of both domains and could not be achieved by either domain alone. This work shows the potential for development of antimicrobials with activity against gram-negative phytopathogens by fusing endolysins with other phage-derived proteins.

We propose a model for the activity of the chimeric endolysin (Fig. 4*H*). We hypothesize that the lipase perturbs the *Psa* outer membrane by degrading a currently unidentified substrate at the cell surface. We theorize that since the lipase is part of the lysis cassette of its parental phage that the protein may serve a similar function to lipases found in the *Bacillus* phage ΦNIT1 (47) and *Mycobacterium* phage Ms6 (59). The lipase from ΦNIT1 degrades bacterial EPS and allows infection of stationary phase bacteria (47), while the lipase from Ms6 was essential for complete lysis of mycobacteria for phage release (59). The lipase in ELP-E10 had activity toward ester bonded β-D-galactosides, which may form part of EPS in *Psa*. Previous structural characterization of *Psa* strain ICMP 18884 EPS has revealed it to contain ⍺-D-rhamnans, ⍺-D-fucofuranose, and ⍺-D-1,4-linked glucans (60); but no genetic determinants of EPS production were investigated. Like *P. aeruginosa*, *Psa* contains *psl* and *pel* operons (61, 62) for EPS production. Genes in these loci may be producing a membrane bound β-D-galactoside that the phage lipase targets to perturb the outer membrane. The full protein from which part24 is derived contains a predicted carbohydrate-binding module similar to those that target 1,4-glucans (58), which could facilitate the lipase in efficiently degrading a substrate produced by *Psa*. Furthermore, hydrolysis of lipids in bacterial EPSs or outer membranes may allow the endolysin to access and degrade the peptidoglycan to elicit bacterial lysis (Fig. 4*H*).

An important feature of our engineered lysin was the specificity toward *Psa* even when combined with citric acid. The specificity might be conferred by the CBD of the jumbo phage modular endolysin in ELP-E10. By contrast, a globular *Psa* phage endolysin combined with citric acid reduced different *P. syringae* pathovars to below the limit of detection (42). Previously, switching of CBDs altered the host range of endolysins against gram-positive pathogens (63, 64). We predict that the strain specificity of ELP-E10 is the result of a combination of the CBD, EAD, and lipase domains as well as differences in the susceptibility of *Psa* and other bacteria to citric acid.

Through the development of an endolysin fusion with specificity toward *Psa*, we have illustrated the power of utilizing a phage protein repository in combination with VersaTile DNA shuffling. Indeed, such a platform could be used to repurpose phage parts from phages not fit for therapeutic application; such as temperate or transducing phages. However, while this work shows promise there are still hurdles to be overcome. For instance, for the chimera ELP-E10 to have practical utility for inclusion in kiwifruit orchard *Psa* management, combination with citric acid or additional engineering would be required to increase antibacterial activity. Our results highlight the potential of phage proteins for engineering endolysin fusions with exogenous activity against pathogenic bacteria of agricultural significance.

## Experimental procedures

### Bacterial strains and growth media

Strains used are listed in Table S5. For cloning and plasmid storage, either *E. coli* TOP10 or DH5α (Thermo Fisher Scientific) were used. For protein expression, either *E. coli* BL21(DE3), LOBSTR, or BL21(DE3) pLysS strains (Thermo Fisher Scientific) were utilized. All strains were grown in lysogeny broth (LB) (1% w/v tryptone, 0.5% w/v yeast extract and 0.5% w/v NaCl) supplemented with either ampicillin (Ap; 100 μg/ml), chloramphenicol (Cm; 25 μg/ml), or kanamycin (Km; 50 μg/ml) as required. Autoinduction media with a terrific broth base (Formedium) was used for protein expression. For bacteria tested in growth inhibition or antibacterial assays, no antibiotic selection was required. Negative selection for constructs made using the VersaTile shuffling cloning methodology (22) was achieved by supplementing 1.5% w/v LB-agar (LBA) with 10% w/v sucrose to counter select any pVTE or pVTD3 vectors still containing *sacB*.

### *In silico* analysis

Phage proteins and domains were identified and annotated using InterProScan (65) and RAST (66). Secondary structure predictions were made using Phyre2 (67) for delineation of domains from phage proteins. The NCBI BLASTn and BLASTp databases were used to identify similar phage sequences (68). Alignment and comparison of the endolysin genes were performed using Geneious alignment with the Blosum62 scoring matrix in Geneious Prime (Version 2021.2.2). Structures were predicted using ColabFold (69). The DALI server (70) was used to find similar structures, which were superimposed on predicted structures using PyMOL (The PyMOL Molecular Graphics System, Version 2.5.2 Schrödinger, LLC).

### Cloning of tiles for VersaTile shuffling

Candidate phage proteins were assessed for compatibility with the VersaTile cloning platform—specifically gene size and absence of SapI and BsaI restriction sites. Domains were chosen from genes displaying high intragenic variability, which is often correlated with host specificity (*e.g.* phage tail fibers) (71). To increase potential expression of chimeric endolysins, the total protein size was limited to ∼100 kDa. Domains from large proteins such as tail fibers and depolymerases were delineated (24) to preserve secondary structure using Phyre2 predictions (67). After curation of appropriate phage parts and endolysins, we designed primers for each part that were compatible with VersaTile (Table S6), so that 3-part endolysin fusions could be produced. Domains of interest were fused by polypeptide linkers in position 2 that were utilized in previous VersaTile fusions (72). Primers were used to amplify the phage parts and clone these into the entry vector (Table S6) to produce tiles for shuffling reactions. Specifically, tiles were prepared by cloning selected genes from phage genomic DNA and gene blocks (Integrated DNA Technologies) (see Table S1 for a list of phages used in this work) using VersaTile compatible primers (Table S6). Each primer contained a BsaI (NEB) recognition site (for variant library construction), a position-dependent tag, a SapI (NEB) restriction and recognition site (for cloning into the entry vector) and ∼20 to 30 nucleotides homologous to the gene of interest. Genes of interest were then PCR-amplified using Phusion polymerase (Thermo Fisher Scientific) and ∼10 ng of applicable phage genomic or gene block DNA. Gene blocks with silent mutations were used to remove SapI or BsaI recognition sites for genes that naturally contained these restriction sites. PCR amplicons were visualized using gel electrophoresis and 1.5% w/v agarose gels supplemented with ethidium bromide. Bands of the correct size were gel-extracted (Cytiva GFX PCR DNA and Gel Band Purification kit), 50 ng of DNA was ligated into 100 ng of the pVTE vector by 10 U of SapI for restriction digest (NEB) and ligation with 15 U of T4 DNA ligase (NEB) in a 20 μl reaction volume containing 2 μl 10X T4 ligase buffer. The full ligation mixture was used for transformation of chemically competent *E. coli* TOP10 or DH5α and plated onto LBA supplemented with Ap and sucrose. Resultant colonies were screened with the primers PF4714 and PF4715. Positive colonies had tile sequences confirmed by Sanger sequencing and stocks of 46 nM were prepared for library construction.

### Variant library production and screening

To produce chimeric *Psa* phage endolysin libraries with 3-part configurations, 2 μl tile mixtures of tiles at relevant positions were added to a 20 μl reaction mixture containing 100 ng pVTD3, 10 U of BsaI, 3.5 U of T4 ligase, and 2 μl 10X ligation buffer. Electrocompetent *E. coli* BL21(DE3) was then transformed with the full ligation mixture and plated onto LBA with Km and sucrose. This produced two variant libraries: PLE had the configuration of phage parts at the N termini, joined by short polypeptide linkers to EADs at the C termini, while ELP had EADs at the N termini and phage parts at the C termini.

Variant libraries were screened by both medium and HT methods. The medium-throughput method has previously been described by Gerstmans *et al.* (22). This method involved picking variant colonies into separate wells of a 96 x deepwell (2.2 ml) plate with 500 μl LB with Km in each well before being sealed with an Aeraseal (Excel Scientific) and grown overnight at 37 °C, with 1200 RPM shaking in a microplate shaker (VWR). Aliquots of 15 μl were taken from each well and used to inoculate 500 μl of autoinduction media with Km and grown at 37 °C with 1200 RPM shaking in a microplate shaker for ∼6 h. The temperature was adjusted to 16 °C and protein expression grown for a further ∼42 h. Cells were pelleted at ∼3500*g* for 30 min at 4 °C, the supernatant discarded and stored at −20 °C. Cell pellets were subjected to chloroform vapor lysis for ∼2 h and then resuspended in 500 μl lysis buffer (pH 7.4, 20 mM Hepes, pH 7.4, 150 mM NaCl, 1 U of DNase I (Roche) and protease inhibitor cocktail (cOmplete mini EDTA-free, Roche)). Plates were incubated at room temperature with 700 RPM shaking in a microplate shaker for 1 h. The insoluble fraction was then separated from the soluble fraction by centrifuging plates at ∼350*g* for 30 min at 4 °C. Aliquots of 50 μl of soluble protein lysate was then added to 150 μl of *Psa* culture with an *A*_600_ of 0.05 (∼10^7^ CFU/ml) and growth monitored by measuring *A*_600_ over a ∼24 h period in a Varioskan plate reader (Thermo Fisher Scientific) at 25 °C with 300 RPM shaking. Growth inhibition was determined after ∼17 h by dividing the *A*_600_ of variant wells by the average *A*_600_ for negative control wells (GFP control lysate added, no growth inhibition expected).

The HT-screening of phage parts that created enzymatically active chimeric *Psa* endolysins involved a halo-based assay (73). In brief, electrocompetent *E. coli* BL21(DE3) pLysS were transformed with ligation mixtures from shuffling reactions. Transformed cells were either plated onto LBA with Km, Cm and sucrose or LBA with Km, Cm, sucrose, 0.1 mM IPTG and 4% crude *Psa* peptidoglycan (prepared as described below). Plates without IPTG and peptidoglycan represented the total library pool, while plates with added IPTG and peptidoglycan determined active variants by the presence of a halo around resultant colonies. Variant libraries were incubated for ∼18 h at 37 °C before being assessed for activity. Active variants were picked onto LBA plates supplemented with Km and sucrose. Crude *Psa* peptidoglycan was prepared by inoculating 600 ml of LB with 6 ml of overnight *Psa* culture (*A*_600_ ∼2) and growing inoculums to stationary phase for ∼24 h. Inoculums were then autoclaved under normal conditions and the resulting debris was pelleted at 4000 RPM for 30 min. The resultant pellets were washed twice in PBS, before being resuspended to a final concentration of 0.4 g/ml in PBS. Resultant colonies from the total and active libraries were individually pooled and plasmid DNA extracted using a ZymoPURE II Plasmid Maxiprep kit.

### Library sequencing and analyses

The pooled EAD-Linker-RBP plasmids (ELP libraries) were sequenced using MinION R10.4.1 flow cells (Oxford Nanopore Technologies) with the SQK-RBK114 to 96 or SQK-LSK114 adapter kits. For samples sequenced using the SQK-LSK114 ligation-based kit, the plasmid pools were first linearized using Ecl136II. The linear DNA was then prepared using the DNA and end-prep and adapter ligation and clean-up protocols. Nanopore reads were demultiplexed and basecalled using Dorado (Oxford Nanopore Technologies) or Guppy (Oxford Nanopore Technologies) with the super accurate (dna_r10.4.1_e8.2_400bps_sup@v5.0.0) model, quality controlled using NanoFilt (74) (using the parameters: -q 8; --length 3000; --maxlength 20,000), then converted to Fasta format using seqtk (github.com/lh3/seqtk). To identify complete chimeric phage protein fusion constructs in the processed sequencing reads, we mapped the expected constituent nucleotide sequences (parts, linkers, and destination vectors) to each read using Bowtie2 (75) (-D 20; -R 3; -N 1; -L 10; -i S,1,0.20; --score-min G,5,8; --ma 2; --mp 2,2 –rgd 4.3 –rfg 4,3 –local; --no-unal; --soft-clipped-unmapped-tlen; --no-hd), then used a graph-based (shortest path) approach to identify which combination of phage parts best matched the read sequence. Nodes represented parts in their expected tile positions (1–3) and edge weights were based on the alignment lengths, scores and edit distances for each part mapped to the read. Only complete chimeric phage protein fusions, where expected parts were identified in all three positions, were included in the final analyses.

### Expression and purification of *Psa* phage endolysin fusions

*Psa* endolysin fusions were purified using the C-terminal His_6_-tag after expression in *E. coli* LOBSTR cells containing appropriate plasmids. For expression, 100 ml or 500 ml of terrific broth autoinduction media, supplemented with Km, were inoculated with either 100 μl or 500 μl overnight culture, respectively, and grown at 37 °C, 180 RPM shaking to midexponential phase (*A*_600_ 0.6–0.8), before moving to 16 °C for further ∼40 h of expression. Cells were pelleted at 4000*g* for 30 min at 4 °C, then stored at −20 °C. Pellets were resuspended in lysis buffer (20 mM Hepes–NaOH, pH 7.4, 150 mM NaCl, 0.6 mg/ml lysozyme 0.02 mg/ml DNaseI, cOmplete EDTA free protease (Roche), and 10 mM imidazole) and lysed *via* sonication (Branson Ultrasonics Sonifier, 50% amplitude, 10 s pulse, 10 s rest, total 2 min, for 2 cycles with a 3 min rest period on ice between each cycle). Lysate was clarified by centrifugation at 10,000*g* for 45 min at 4 °C, and the soluble fraction was syringe-filtered (0.45 μM).

ELP-E10 was purified *via* immobilized nickel affinity chromatography using a 5 ml HisTrap HP (Cytiva) column that was equilibrated in binding buffer (20 mM Hepes–NaOH, pH 7.4, 150 mM NaCl, 150 mM NaCl, 20 mM imidazole). The column was washed with five column volumes of binding buffer supplemented with 50 mM imidazole, and the protein eluted using a gradient of elution buffer (binding buffer containing 500 mM imidazole) using a FPLC system (ÄKTA Pure, Cytiva). The His_6__-_tag remained attached to purified proteins. Fractions containing the fusion endolysin, as identified using Coomassie Blue stained Bolt 4 to 12% Bis-Tris SDS-PAGE gels (Invitrogen), were pooled and concentrated with a centrifugal concentrator (Cytiva Amicon; 30 kDa molecular weight cutoff (MWCO)) and purified further by SEC on a HiLoad 16/600 Superdex 200 (Cytiva) column equilibrated in SEC Buffer (20 mM Hepes–NaOH pH 7.4, 150 mM NaCl, and 10% w/v glycerol). Elution peak fractions at ∼55 kDa were analyzed by SDS-PAGE, pooled, and concentrated to ∼1 mg/ml with a 30 kDa MWCO centrifugal concentrator.

Variants and individual domains of ELP-E10 were purified by batch column purification. In brief, following lysis, clarified lysate was equilibrated with 1 ml of Ni-NTA agarose slurry (Protino) for 30 min on a Hula mixer at 4 °C. Slurry mixtures were then transferred to Poly-Prep chromatography columns (Bio-Rad); once resin had separated, columns were washed with 2 × 5 ml 50 mM imidazole washes. Proteins were then eluted in 5 × 500 μl fractions using elution buffer. Eluted fractions were then pooled, and imidazole was removed by exchange into SEC buffer using PD-10 desalting columns (Cytiva). Proteins were concentrated with 10 or 30 kDa MWCO centrifugal concentrators, filter sterilized with 0.22 μm filters and then used in assays. Protein concentrations were determined using the Qubit Protein Assay Kit (Invitrogen).

### Mass spectrometry

Mass spectrometry was used to confirm the identity of purified proteins. Briefly, protein samples were mixed with 4× SDS loading dye (40 mM Tris–HCl (pH 6.8), 40% glycerol, 4 mM EDTA, 2.5% SDS, and 0.2 mg/L bromophenol blue), boiled for 10 min and separated on a 4 to 12% SDS-PAGE gel (polyacrylamide gel electrophoresis). The gel band was excised and subjected to automated in-gel tryptic digestion following reduction and alkylation of cysteine using a liquid-handling robotic workstation. The resulting tryptic peptides were dried in a speedvac and resolubilized in 5% [v/v] acetonitrile, 0.1% [v/v] formic acid. Liquid chromatography-mass spectrometry based protein identification was performed using an LTQ-Orbitrap XL mass spectrometer inline coupled to an Ultimate 3000 nano-flow uHPLC-system (Dionex Co., Thermo Fisher Scientific). The data were then processed through the Proteome Discoverer software (Thermo Fisher Scientific) using the Sequest search engine mode. The data were queried to an in-house FASTA database containing the sequence of proteins of interest. In the search setting, allowance was made for the deamidation of the asparagine and glutamine and carbamidomethyl was selected as a static modification.

### Muralytic activity assays

The peptidoglycan degrading ability (muralytic activity) of endolysin fusions was evaluated using an assay of *P. aeruginosa* spheroplast turbidity reduction (76). Briefly, *P. aeruginosa* PAO1 cells were grown at 37 °C, with 180 RPM shaking, to an *A*_600_ of ∼0.6. Cells were pelleted at 4000*g* for 15 min at 4 °C and resuspended in 0.05 M Tris–HCl (pH 7.4) saturated with chloroform. Cells were incubated for 45 min with 80 RPM shaking at 25 °C. Spheroplasts were then pelleted (4000*g*, 15 min, 4 °C) and washed and resuspended in 80 mM KH_2_PO_4_/K_2_HPO_4_ buffer (pH 7.3) to an *A*_600_ of ∼1. Protein aliquots (30 μl) of varying concentrations were added to spheroplasts (270 μl) in a 96-well plate and incubated at room temperature with no shaking in a VICTOR Nivo plate reader (PerkinElmer, Inc) and the change in *A*_600_ was measured every minute for 3 h. Each condition was repeated in technical triplicate with data plotted as the mean ± SEM. The activity of each protein (measured in units/mg) was calculated as previously described (76).

### Antibacterial activity assays

Unless otherwise mentioned *Psa* 18884 was used to assess antibacterial activity. Cells were grown to exponential phase *A*_600_ 0.4 to 0.6 and then pelleted at 6000*g*, 10 min, 4 °C, and washed and resuspended in 20 mM Hepes–NaOH pH 7.4 and 150 mM NaCl, then further diluted 1:10 to a final density of ∼10^6^ colony-forming units (CFU)/ml. Treatment combinations were made at desired concentrations in a total volume of 100 μl in 20 mM Hepes, pH 7.4, 150 mM NaCl, 10% glycerol, and then added to 100 μl of bacterial suspensions in a 96-well plate and incubated for 1 h at 25 °C, with shaking (160 RPM). Following incubation, cells were serially diluted to 10^-7^ in 20 mM Hepes, pH 7.4, 150 mM NaCl and plated onto LBA. Following incubation, CFU/ml was enumerated and log_10_ reduction calculated. The FICI for ELP-E10 with EDTA or citric acid, was calculated using the following formula: FIC_A_ + FIC_B_ = FICI, where FIC_A_ equals the minimum inhibitory concentration (MIC) of compound A in combination divided by the MIC of compound A alone and FIC_B_ equals the MIC of compound B in combination divided by the MIC of compound B alone. The MIC was determined as the concentration required for a 0.6-log (75%) reduction in CFU/ml. The FICIs were interpreted as follows: synergy: FICI of ≤0.5; additivity: FICI of >0.5 to ≤1; no interaction (indifference): FICI of >1 to ≤4; antagonism: FICI of >4^37.^

### Site-directed mutagenesis

The catalytic activity of part24 was abolished through the mutations D623A and H626N using 20 bp overlapping primers (Table S6) and Q5 polymerase (NEB). The coding sequence of part29 was sequentially mutated with the substitutions E115A and Y197F using the same methodology as used for part24. Resulting PCR products were treated with DpnI to remove PCR templates, and Gibson assembled with NEBuilder HiFi master mix (NEB) to ligate PCR products together to form plasmids with mutant domains.

### Hydrolase activity assay

To assess the substrate profile of endolysin fusion proteins, particularly the putative lipase (part24), hydrolase activity was screened using a previously established method (77) with a panel of 4-MU based fluorogenic substrates. Model substrates included phosphate, lipid chains of various length, and various sugar groups. Reactions contained 160 nM endolysin fusion protein and 10 μM 4-MU substrate, in 1× PBS buffer pH 7.4, 0.01% Triton X-100 at a total volume of 60 μl. Reactions were performed in a Greiner 96-well flat-bottomed plate that was incubated at 37 °C. Fluorescence (λ_ex_ = 365 nm, λ_em_ = 455 nm) was monitored on a CLARIOstar instrument at 62 s intervals for 60 min. Measurements were performed for six replicates using two different preparations of an endolysin fusion protein. The rates of reactions were determined for each replicate as a change in relative fluorescence units per minute.

## Data availability

Data supporting this study are included within the article and/or supporting materials.

## Supporting information

This article contains supporting information.

## Conflict of interest

Dennis Grimon is co-founder of Obulytix, Yves Briers is co-founder and scientific advisor for Obulytix and Diana Gutiérrez is CSO of Telum therapeutics. The other authors declare that they have no conflicts of interest with the contents of this article
