# Supplementary material for: Engineering an antimicrobial chimeric endolysin that targets the phytopathogen Pseudomonas syringae pv. actinidiae
Source: J Biol Chem. 2025 May 9;301(6):110224. doi: 10.1016/j.jbc.2025.110224 (PMC12173070; doi:10.1016/j.jbc.2025.110224)
Supplement: Supporting information [file mmc1.pdf]

SUPPLEMENTARY INFORMATION

Engineering an antimicrobial chimeric endolysin that targets the phytopathogen *Pseudomonas syringae* pv. *actinidiae*

Suzanne L. Warring[1,2,3†*], Hazel M. Sisson[1,3,4†], George Randall[5], Dennis Grimon[6], Dorien Dams[6], Diana Gutiérrez[6], Matthias Fellner[5], Robert D. Fagerlund[1,2,3,4], Yves Briers[6], Simon A. Jackson[1,2,3,4] and Peter C. Fineran[1,2,3,4*].

[1]Department of Microbiology and Immunology, University of Otago, P.O. Box 56, Dunedin 9054, New Zealand.
[2]Maurice Wilkins Centre for Molecular Biodiscovery, University of Otago, P.O. Box 56, Dunedin 9054, New Zealand
[3]Genetics Otago, University of Otago, P.O. Box 56, Dunedin 9054, New Zealand.
[4]Bioprotection Aotearoa, University of Otago, P.O. Box 56, Dunedin 9054, New Zealand.
[5]Department of Biochemistry, University of Otago, P.O. Box 56, Dunedin 9054, New Zealand.
[6]Department of Biotechnology, Ghent University, Ghent, Belgium.

Present addresses: SW: Seperex Nutritionals Ltd. Centre for Innovation Otago, 87 St Davids Street, Dunedin 9016, New Zealand. SJ. Biomedical Sciences, Division of Health, University of Waikato, Private Bag 3105, Hamilton 3240, New Zealand.

† These authors contributed equally to this work
*For correspondence: suzanne.warring@otago.ac.nz, peter.fineran@otago.ac.nz.

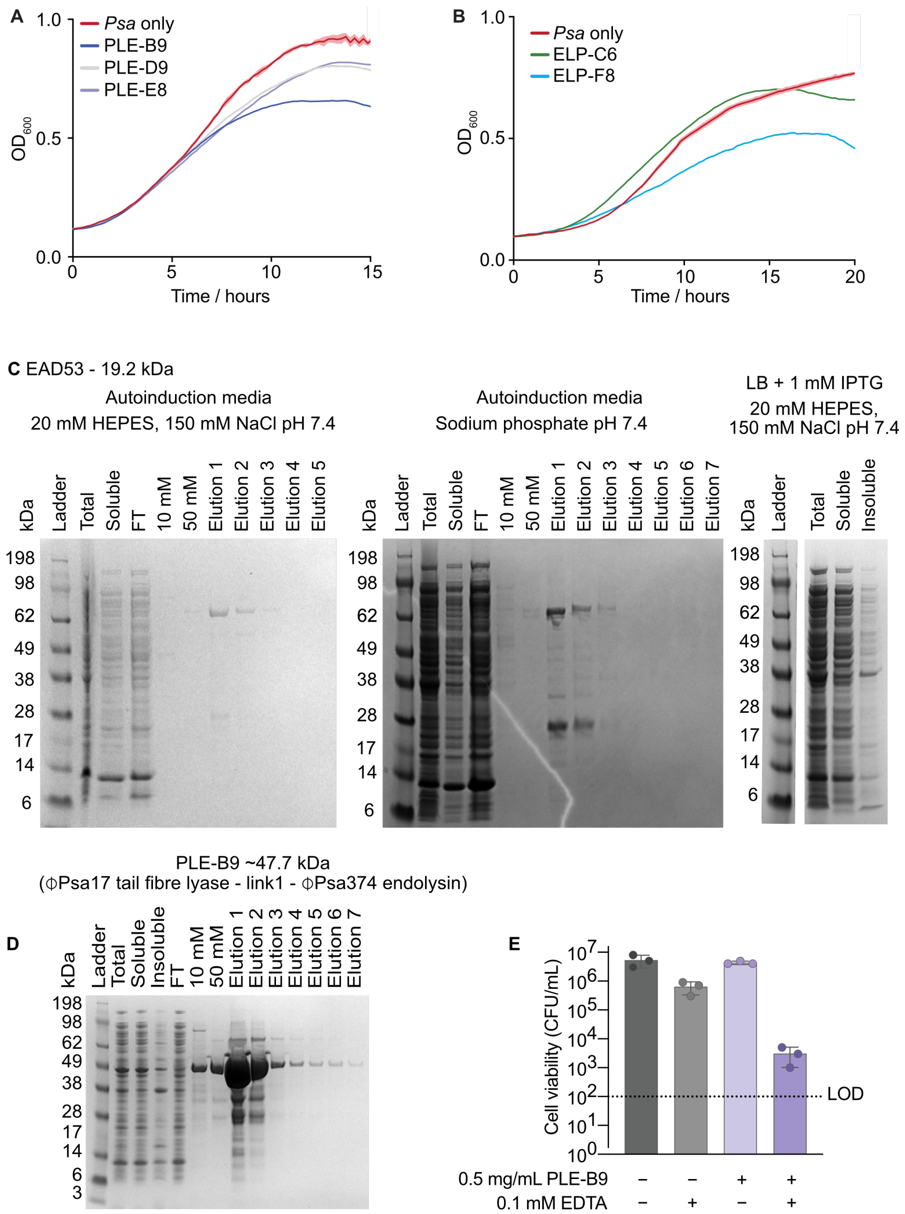

Figure S1: Supplementary data for medium through-put library screening of endolysin chimera variants. A. & B. Representative growth inhibition data for lead PLE and ELP variants, data is presented as single replicates from medium through-put assays shown in Figure 1A. *Psa* growth data is presented as the average of 3 replicates with error presented as the SEM. **C.** Expression and purification trials of EAD53 with different induction methods and buffers. **D.** Coomassie blue stained SDS-PAGE gel of purification of variant PLE-B9 from a 100 mL expression. **E.** Cell viability of *Psa* 18884 WT treated with 0.5 mg/mL PLE-B9 with and without 0.1 mM EDTA. Data is presented as the mean +/- SD and individual data points are biological triplicates.

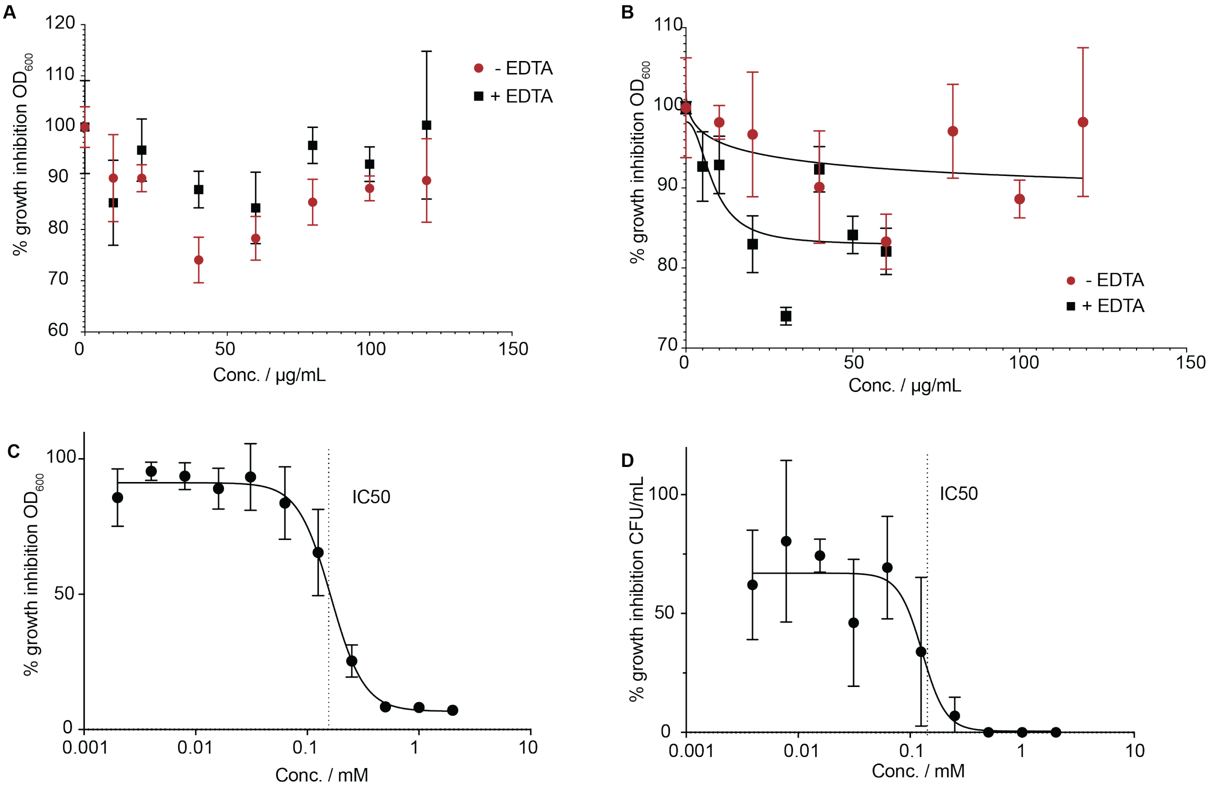

Figure S2: IC_{50} data of lead variants and EDTA. A. IC_{50} data from OD_{600} of increasing concentrations of ELP-D9, with and without 0.1 mM EDTA grown with WT *Psa*. **B.** IC_{50} data from OD_{600} of increasing concentrations of ELP-E10, with and without 0.1 mM EDTA with WT *Psa*. **C.** IC_{50} data determined from OD_{600} of increasing concentrations of EDTA grown with WT *Psa*. **D.** IC_{50} data determined from CFU/mL data of EDTA added to WT *Psa*. Data has been normalized the growth control without the addition of EDTA. Data is presented as the mean +/- SD and each data point are biological triplicates.

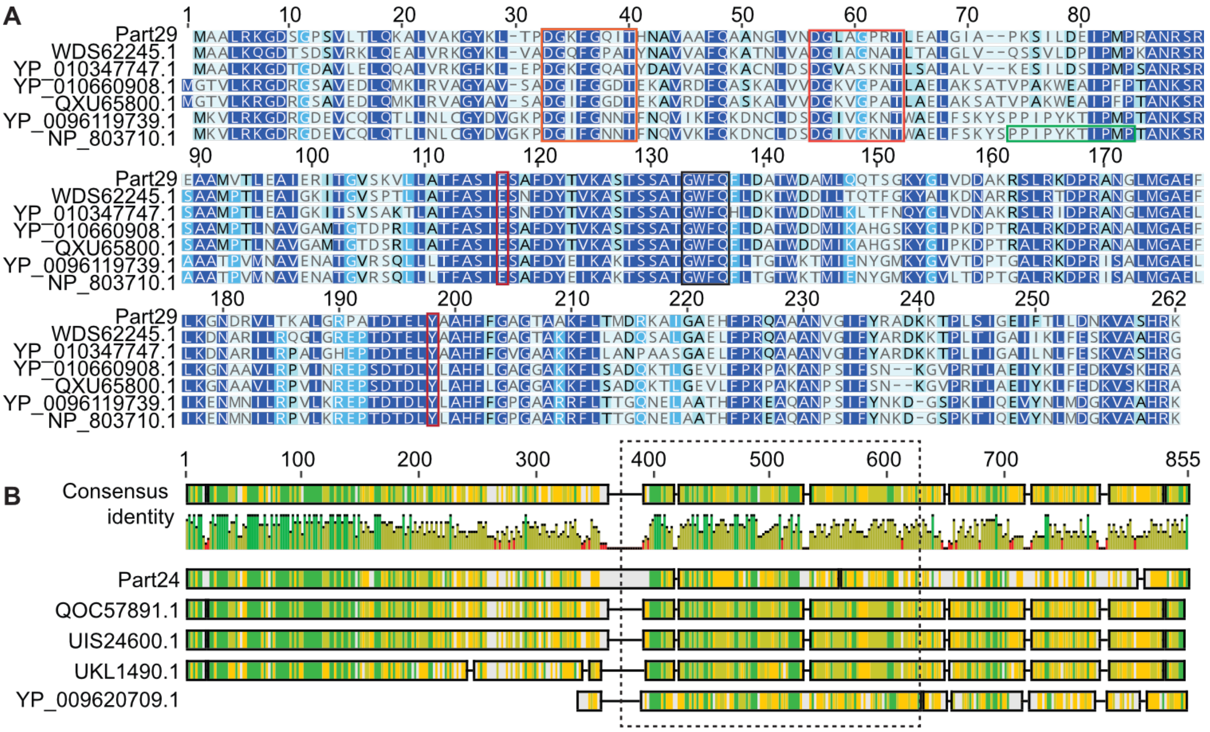

Figure S3: Protein sequence alignments of part29 to similar proteins. A. Protein alignment of the ELR-E10 variant endolysin domain (part29) with endolysins from related Pseudomonas phages: D6 (WDS62245.1), ΦPsa21 (YP_010347747.1)[1,2], PP933 (YP_010660908.1), PA90 (QXU65900.1), SL2 (YP_009619739.1)[3] and PhiKZ (gp144, NP_803710.1)[4]. Conserved residues are indicated by dark blue, while residues with <60 % similarity are shown in white. Orange boxes indicate conserved repeat regions, and the green box indicates a proline-rich region in gp144 (NP_803710.1) in the peptidoglycan domain. Red boxes show active site residues in transglycosylase domains, while the black box highlights a transglycosylase superfamily 1 motif. The sequence alignment of part29 and gp144 shows similar transglycosylase domains including active site residues E115[5] and Y197[6] and the weakly conserved GXFQ transglycosylase family 1 motif[5] (red boxes). The peptidoglycan domain of part29 contains the expected repeated residues (D1, G2, G5 and T8, orange boxes), but not the same proline rich linker region as gp144 (green box for gp144)[5]. The two missing prolines in part29 relative to gp144 may account for the deviation in secondary structures of the peptidoglycan binding domains as proline is a rigid amino acid that affects protein folding[7]. **B**. Protein alignment of part24, the phage part domain of ELP-E10, from a *Psa* siphoviridiae phage N2 with orthologous lipases/GDSL hydrolases from related *Pseudomonas* phages (accession number): phiK7B1 (QOC57893.1), ZY21 (UIS24600.1)[8], hairong (UKL14940.1)[8] and nickie (YP_009620709.1). Dark green shows 100 % similarity, olive shows 80 - 100 % similarity, yellow shows 60 - 80 % similarity, and white shows <60 % similarity.

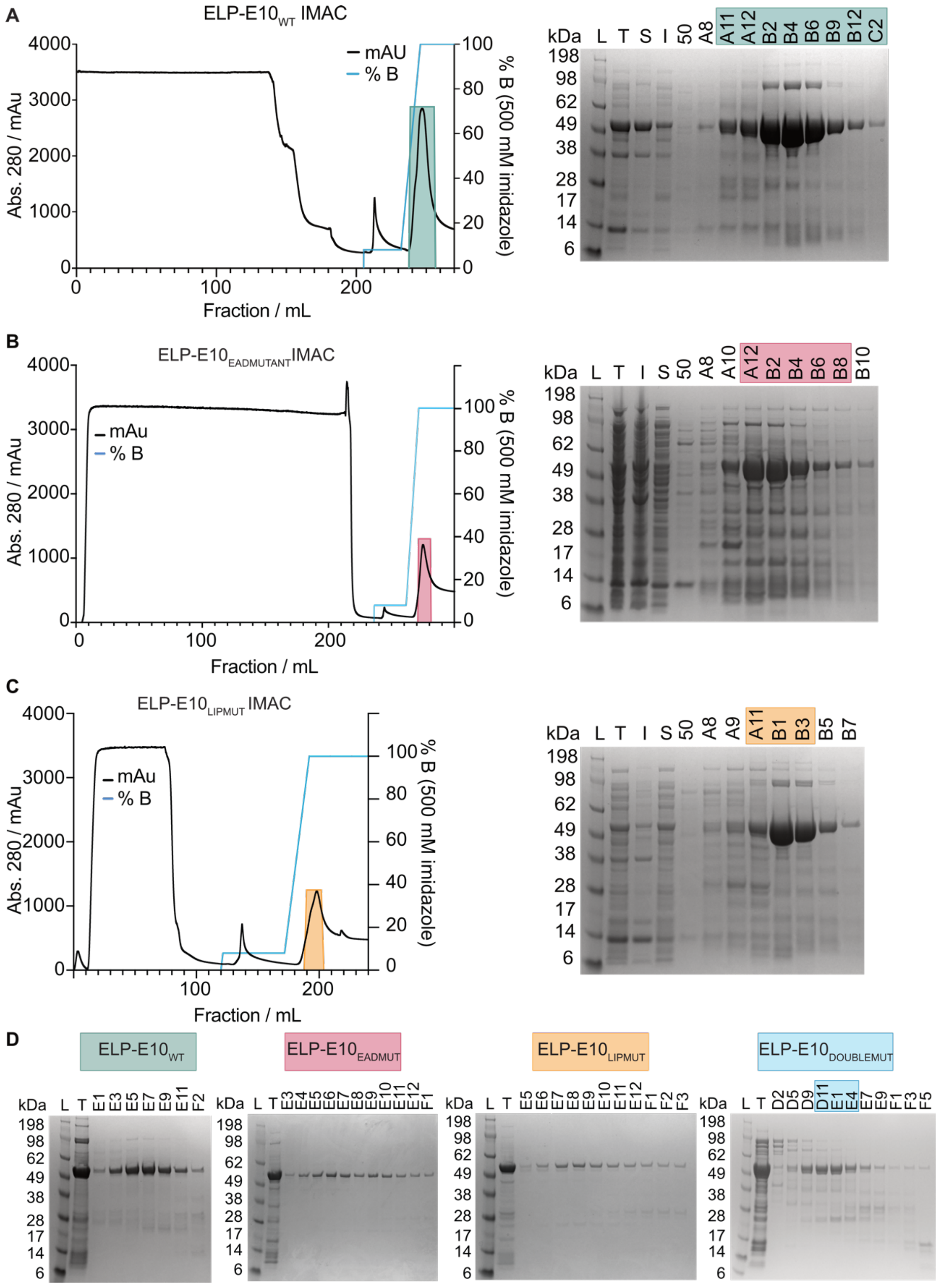

Figure S4: Purification of ELP-E10 WT and point mutants. A-C. NiNTA IMAC traces with 4-12 % Bis-Tris SDS-page gel showing fractions collected of **A.** ELP-E10 WT **B.** ELP-E10$_{EADMUT}$ and **C.** ELP-E10$_{LIPMUT}$. **D.** SDS-PAGE gels of SEC elutions containing ~55 kDa proteins subsequently collected and concentrated for muralytic activity and antibacterial testing. For the ELP-E10$_{DOUBLEMUT}$, fractions collected are indicated by the blue box.

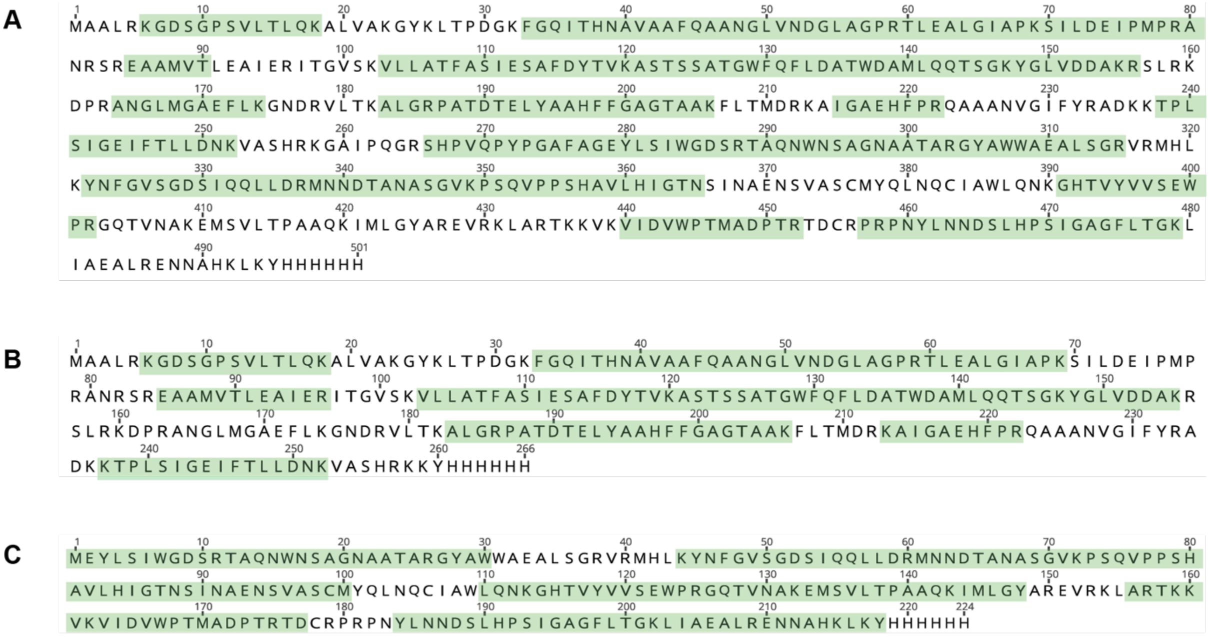

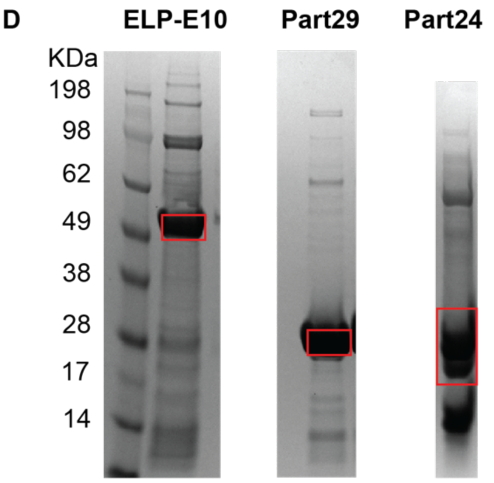

E

	ELP-E10	Part29	Part24
**Coverage**	70.26	79.32	84.38
**# Unique peptides**	66	61	102
**# PSMs**	412	629	268
**MW / kDa**	54.5	28.7	25
**calc. PI**	9.7	9.72	9.55

Figure S5: Mass spectrometry data of proteins. A. Identified peptides in ELP-E10. **B.** Identified peptides in part29, an endolysin derived from a *Psa* jumbo phage. **C.** Identified peptides in part24 a lipase derived from a *Psa* siphovirus. For **A** – **C** green boxes represent peptides identified in analysis with high confidence. **D.** 4-12 % Bis-Tris gels used for mass spectrometry analysis, regions of gels extracted and used for analysis are highlighted in red. **E**. Mass spectrometry results found for each protein. PSM stands for peptide spectrum matches.

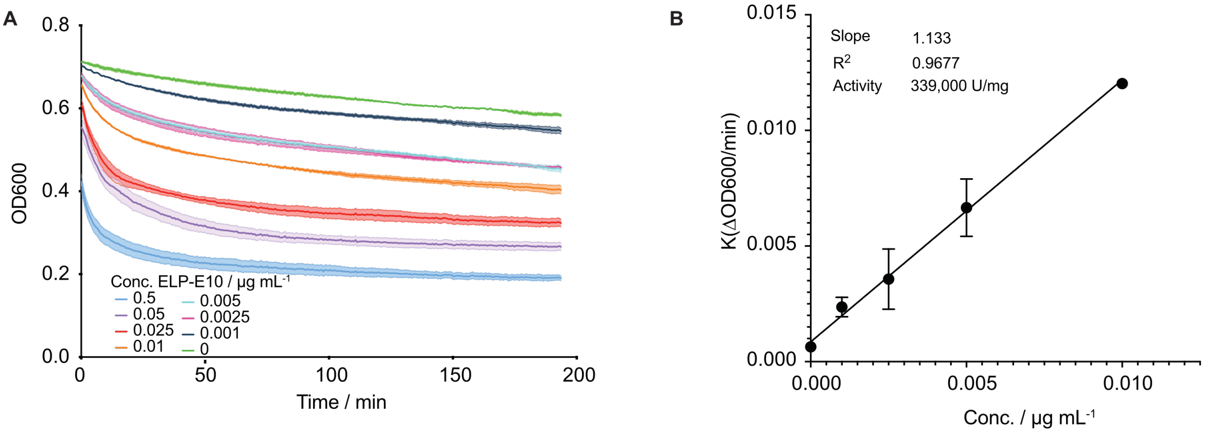

Figure S6: Muralytic activity of ELP-E10. A. Decreases in OD_{600} of PAO1 spheroplasts exposed to varying concentrations of ELP-E10 over a ~190 minute period. Data is presented as the mean +/- SEM, each concentration of ELP-E10 was repeated in technical triplicate. **B.** Linear regression fit of K (ΔOD_{600}/min) determined from raw OD_{600} data using an activity calculator developed by Briers *et al.*[9] and plotted against ELP-E10 concentration to determine specific activity of ELP-E10. Data is presented as the mean +/- SD derived from technical triplicates.

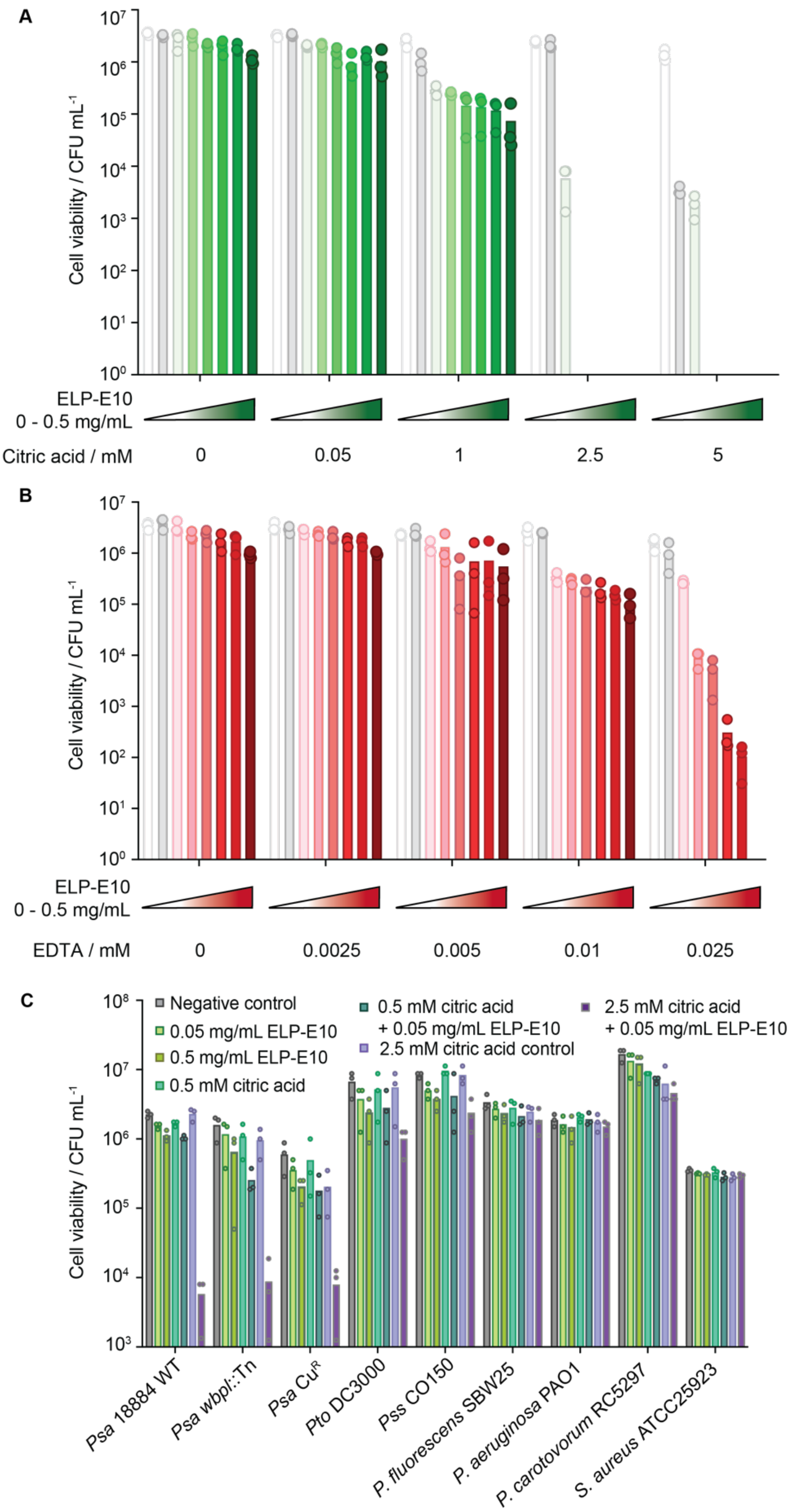

Figure S7: CFU/mL antibacterial data used for Figure 2. A. Chequerboard assay showing antibacterial activity of ELP-E10 against *Psa* 18884 with varying ELP-E10 and EDTA concentration. **B.** Chequerboard assay showing antibacterial activity of ELP-E10 against *Psa* 18884 with varying ELP-E10 and citric acid concentration. **C.** CFU/mL of various bacteria treated with 0.05 or 0.5 mg/mL ELP-E10 and 0, 0.5 or 2.5 mM citric acid. Data is presented as the mean and each data point is a biological replicate. *Psa* Cu^{R} represents copper resistant *Psa* ICMP 246510.

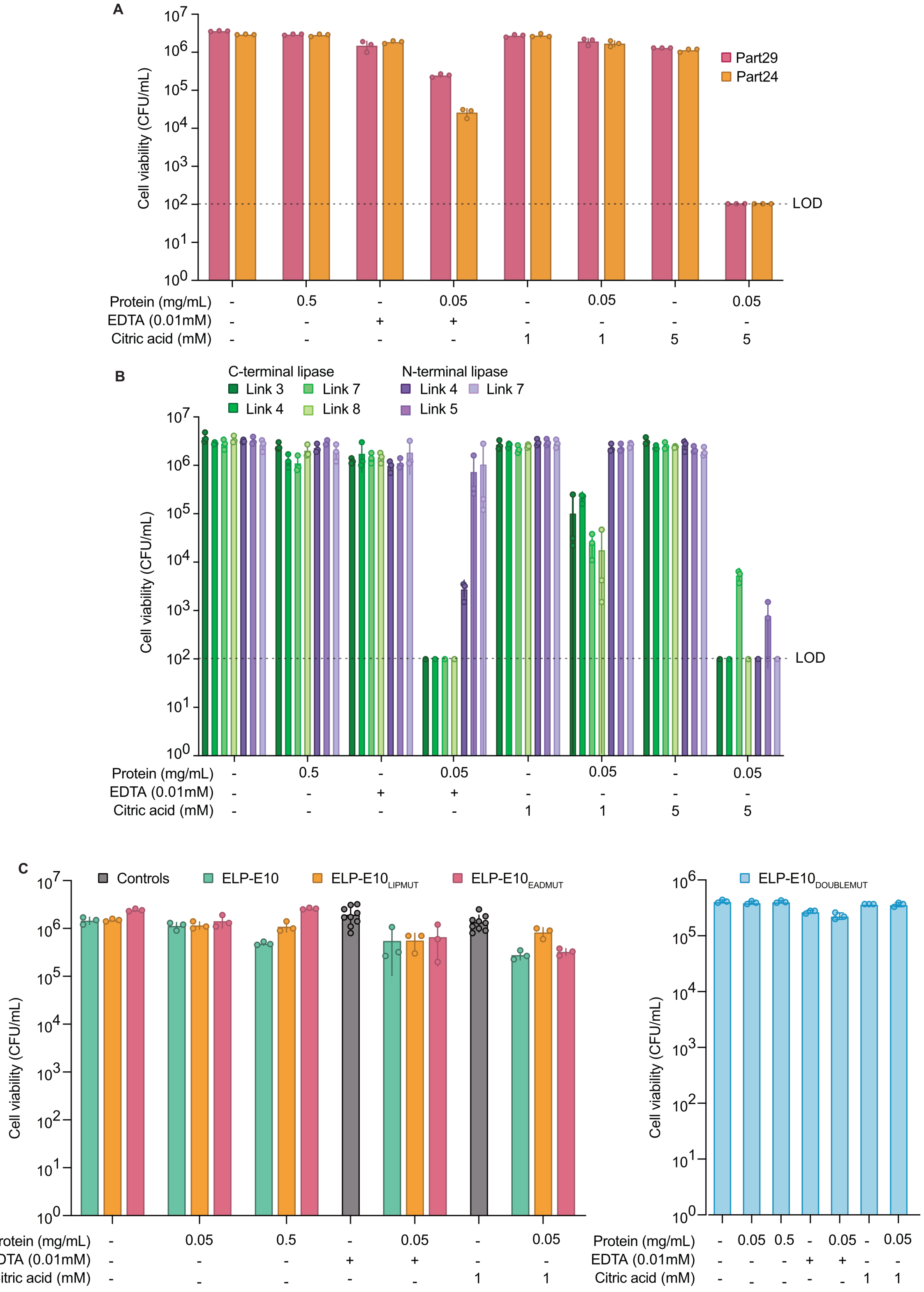

Figure S8: CFU/mL antibacterial data used for Figure 3&4. A. Antibacterial activity of ELP-E10 individual domains against *Psa* at different protein, citric acid and EDTA concentrations. **B.** Antibacterial activity of ELP-E10 variants with differing configurations and linkers in the presence of varying citric acid concentrations and 0.01 mM EDTA against *Psa*. **C.** Antibacterial assay against *Psa* WT of ELP-E10 and mutants alone and combined with chemical permeabilizers. Data is presented as the mean +/- SD and individual data points are biological replicates.

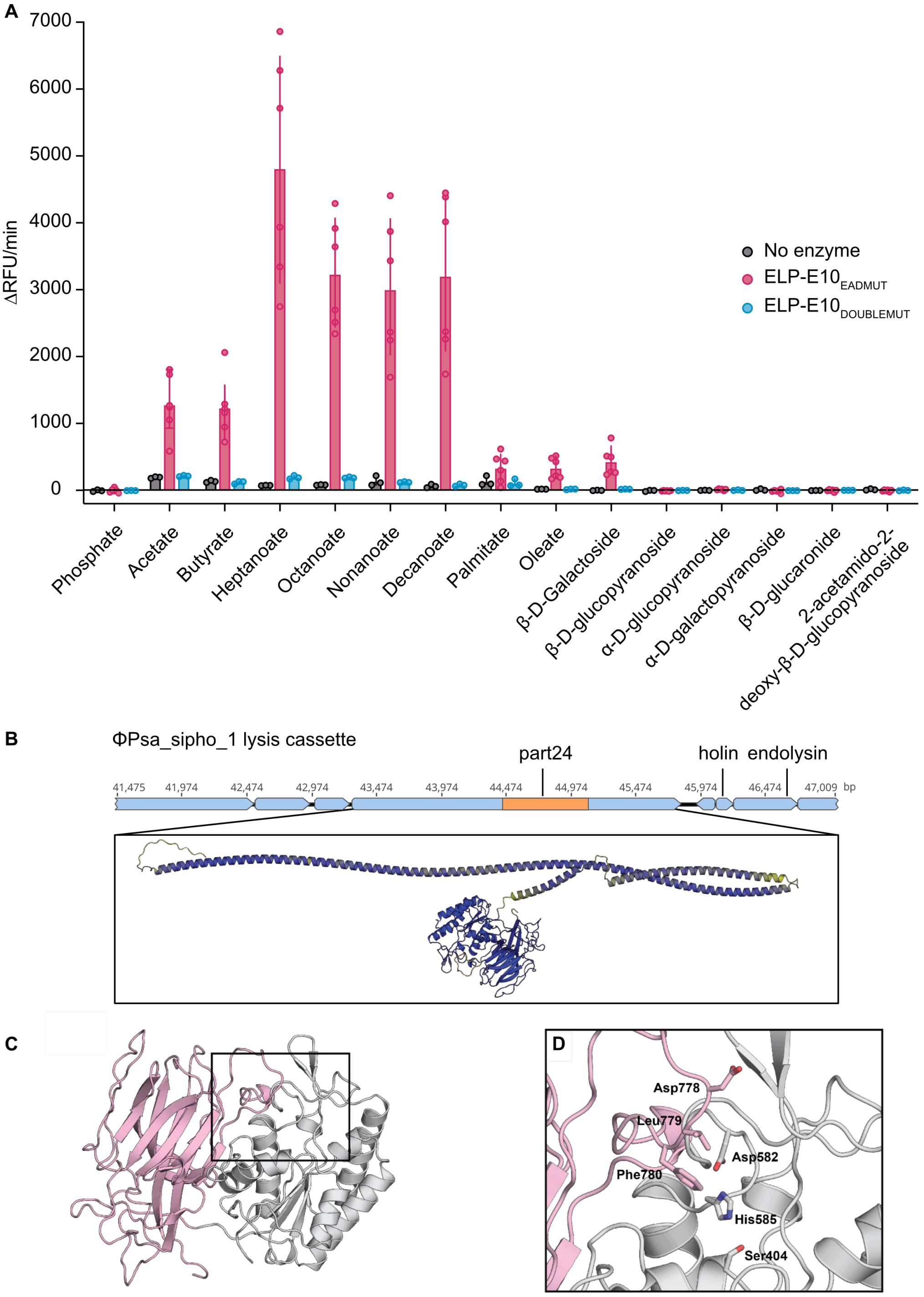

Figure S9: Hydrolase activity and structural characterization of the phage lipase. A. Biological replicate data of lipase activity of ELP-E10$_{EADMUT}$ and ELP-E10$_{DOUBLEMUT}$ against 4-MU model substrates with lipid chains of various length and various sugar groups. Controls are with no enzyme and phosphate only. Data is presented as the mean +/- SD and individual data points are technical replicates. **B.** Section of ϕPsa_sipho_1 genome showing position of holin and endolysin downstream of the gene from which part24 is derived (orange) with an AlphaFold3 model of the full-length hydrolase beneath. The structure is coloured by pLLDT confidence values, yellow being least confident, to blue being most confident. **C.** Cartoon representation of the hydrolase domain that consists of part 24 (white), and the carbohydrate binding domain not included in part 24 (magenta). **D.** Active site of the hydrolase, inside the box in panel **C.** Side chains of the catalytic triad (Ser404, His585, Asp582), and residues of a loop from the carbohydrate binding domain that protrude over the hydrolase active site (Asp778, Leu779, and Phe780) are labelled.

Table S1. Phages used for cloning of EADs and RBPs to produce variant libraries.

Strain	Morphotype	Reference
ϕPsa374	myovirus	[1,19]
ϕPsa315	myovirus	[1,19]
ϕPsa17	podovirus	[1,20]
ϕPsa21	myovirus	[1,21]
ϕPsa_jumbo	myovirus	unpublished
ϕPsa_myo_1	myovirus	
ϕPsa_sipho_1	siphovirus	
ϕPsa_sipho_2	siphovirus	

Table S2. Tile amino acid sequences of domains used in this study.

Tile name	Amino acid sequence
EAD50	MLTEIDYKQAAALLGVEPACVKAVTKVESRGSGFLPSGEPVILFERHWMYKLLKAKT GKEPELNEVCNPKAGGYQGGAAEHIRLNTAVMIDRECALQSASWGLFQIMGFHWK ALGFESVQQFINAQYKSEATQLDTFVRFLKINPGMLTALRTKDWAKFAKLYNGPNY AINKYDTKLAAAYASFI
EAD51	MLTEIDYKLAASLLGVEPACIKAVTKVESRGSGFLPSGEPVILFERHWMYKLLKAKT GKEPELSEVCNPKAGGYQGGAAEHIRLNTAVMIDRECALQSASWGLFQIMGFHWK ALGFESVQKFINQQYRSEAGQLDTFVRFVKLNPGMLAALKAKDWAKFAKLYNGPN YAINKYDEKLAAAYASFI
EAD53	MELNKKVVAAGFVGAITAAAAFIGPEEGLSLKPYQDIGKVWTVCYGHTGTDVIRNKY YTQAACDALFRSDLWVAMSGVLRNTPGVTLPEPVLVSFTSFVYNVGENKFKGSTA RTLLVQGKFEEACHQIPRWKFAAGLDCSVRDNNCYGVWSRRLREEAYCMSAFK
part19	WEQVPMPTSKDAKGSRKTLEVVSQMTGVPIEYLMVFCALESNFDWTVKAGAGGS ATGWFQFINSTWDWVIGQHSAKYGLPADVGRRLRLDPRVNGLMGGEYIKYSMNVI KKGTGKDPTDIDLYLAHFLGPGTAVKWIKMPKTTIGSTAFPKEARANPSVFTDKRTG AQRTLGQIEQSFDERMAKYR
part20	MEVVAQMVGVPVQYLMIFCAMESGFDWTIKAGAGGSATGWFQFINSTWDWMISQ HSQKYGIPADSGRRLRLDPRINALLGAEYMKYSMGVIKKQTGKDPTDIDIYLAHFLG PGTAVKWIKLPKNTIGSSAFPKEARANPSIFTD
part28	MAALKKGDTGDAVLELQQALVRKGFKLEPDGKFGQATYDAVVAFQKACNLDSDGV ASKNTLSALALVKESILDSIPMPSANRSRSAAMPTLEAIGKITSVSAKTLATFASIESN FDYTVKASTSSATGWFQHLDKTWDDMLKLTFNQYGLVDNAKRSLRIDPRANGLMG AELLKDNARILRPALGHEPTDTELYAAHFFGVGAAKKFLLANPAASGAELFPKQAAA NVGIFYARDKKTPLTIGAILNLFESKVASHRG
part29	MAALRKGDSGPSVLTLQKALVAKGYKLTPDGKFGQITHNAVAAFQAANGLVNDGLA GPRTLEALGIAPKSILDEIPMPRANRSREAAMVTLEAIERITGVSKVLLATFASIESAF DYTVKASTSSATGWFQFLDATWDAMLQQTSGKYGLVDDAKRSLRKDPRANGLMG AEFLKGNDRVLTKALGRPATDTELYAAHFFGAGTAAKFLTMDRKAIGAEHFPRQAA ANVGIFYRADKKTPLSIGEIFTLLDNKVASHRK
Linker 3	LSRFFHAEL
Linker 4	VFNQRKEHKGYMLA
Linker 5	IPQGRSHPVQPYPGAF
Linker 6	PAVPPP
Linker 7	EAAAKEAAKEAAK
Linker 8	AGYGKAGGTVTPTPNT
Linker 9	EAAAKEAAAKEAAAK
part1	MSAENLIFELSDKAQRYLSVGIVSAIGGVASYLYEHVKGERDFSFISFITMIFLAFFVG NVLGEFIPHDMQSRDGILMVAGFSSWPILDALKSNGKKIADAVLSRLLKIFK
part2	MIETDIADIIRTQFRIDMSDIHTAIPCKVVNVYQNMENQKVDVIPSVNQLLKDGTGEE GMQILGVPIIFPGSAHTLMSFPINPGDTVWVMFSERSMDNFKIGSGEPTTANDYRKF SDQDAVAIPGLFPFGRSPNSPQVRKFAHEPNRDLCIAHNIGSGTEVNILLKQNGDLII NTESAVTVNCKTGVMNATESYTINTPTMNINASTTNWTGNIIHTGNYTMTGQATFN GVLFDTHFHSGVTPGTGNSGPVAG
part5	MPGISRAGIDSAGGVIQPTQRIATINGAPIATVGASVTGHGSGAHAGPVMVQGSVLF TINGIAVVLAGMRASCNDTASGDPKHTCSA
part6	MPGITRVGIDTAGGVIQGPGAPLVTINGAPVSLVGDSVTGHGSGSHASAVMVQGSA IFTVNGIPVVLAGMRASCNDQATGDPKHTCFA
part8	NTSGNVSAVNLNASGTITGAYITSTGNVNAAGTFTGSQVSVSGGVTAANGSGAVTI SAGVVSIKSTSAGANAHVWFYDNVGNTRGIIYAQQSNTITLQAGGAVCANFSPSGG ASFNLTSTSGIENNGRVRSLKNGVPVNGSMYANCHYMAETADGTPPAYSWHRGG SYALAVWLTNGNELQMMGSDGVSREIVNNSNLPAWNNSLYANGNQNIGSYAFARN GGGTVGFGGAQSGSVLSPGSTGANAGALSGTWICLGYAGGGNHTLWIRTS

Tile name	Amino acid sequence
part9	MTFSVHGSATGGYAVGPVENAFEGVDLAAAQAARDAYFASNPTKLAAYDSNPFYLI RLTYGATTNAEYRMGGQWIDYTPFLQGLPGEVASLANVPVGELPYKTLDGTFAGS RMRVLDDGSLLAPPGFGVESGSVKFGDVLLLSEAAGFLSISNLINERPYTILDYYTPR DAASAVPTAFMASAPAFGFVAQPDDSVNMTDEPLLFDYTIQNTSRTYSLFMRAFAA MSNVRIKITQISNGVALKYLPSKEAWETEEGGLEWIIGDNTFDFGDTPLIFNTGQNIR FEIRATTMSLKGNASGIPYFGGSDQLGEFRDVLMADQYTATDVRDKLATLTGTNRL PMSAIKDGVVSVAGRLGAIVLTASDVAGLSTVATTGAYSSLSGLPFIPTNTSQLTNG ANFITAAQAPVQSVQGYTGAVVLARSDIGLGNVDNTSDINKPTSTAQQTSIDVKMSQ HNAAVDPHPQYTTVAEASAAAPIQSIVNGRGITASTLTGVTTVGSTEFPLVFNGSGA ASSPKIWYGTATSDVNGVFIVDISSAGFTLAPTVTVSATLTTVTLTDRAWATLASVTA TTASGYTLRGINLSGNGATVRLAPNTTVNIMAIGI
part12	SDLPNAQAARNNLGLGNLSTRNVFGVPGDLDFTISKTSGGYTNLPNGLIMQWGQG PALGDDQGTTVGLFVAMNVVSVQVTGIGAATNGIGPGFLTQGWTTTSFQVYNNYN SGPSRPFSWLAIGYV
part13	ATTPKTVRTYALDGTKKDFTIPFEYLARKFVVVTLIGATRRELILNTEYRFTTNTTITTT KAWGPADNFDLIEIRRLTSATERLVDFADGSILRAYDLNISQVQSLHIAEEARDLTAD TIGVNNDGDLDARARKIVNLADGVNDGDAVNMRQQRQWAGSALNSANASAASAQ ASEASRQASLAQAQASAASAGQSSTYATNSQNSASNSQTSRLASEAARDASITAR DLSQAWASK
part14	KKVEINTGWEWNSDVIAVDGFRSRSSSSIDFNAQLNGRLGASFTGSLSVGGTTSLQ NTTVAGLTVNTGAQFKNGNVQVFAPKASDNAHVWFYNPDGATRGILYAGTDKTVR MQAGEVVCAAFSPDGGTTLNNISAGNVTARGAAYTQGGIENNGRVASYRNGVGAN ASMYTNCHFMAQTDDGTAPAYGFHRGGSYALALYLSGNSLFMMDSAGLNREVLN NTNLGSWMVSMGEQGVGSYAFCRSSVTATY
part18	MNPVLHTLPLDWSGESPDNRVPGEWHDLADQYDLPFRVVTLRHGYFFADSVKIMD GNGYELKKDRDFQCIAFNGDAAEKIAEPVCGVIVIVNPAVHYIIKVDAQMLGGEYCS MGRSIAEAATALQNTTRKIHWSNITGKPDDYRPNGHLHALWDLYGFTPQVLQLKRM TKGFELMVQKDFDGLQAQFDQSMLGMENLLDDVEAQLTAHIADVVSNPHRDSKAK LIPSLGNVQNQPVATESEARLSDTSVMNRYATPWSMAVSINTNFWARFNEHIGNID NPHNVTAAQLNVYTIGEWNTLSQLYVPLHATTRSSNQIYGMTPSGYYNLIRTGNAV GSIDITASRLNPGRFSSTNYWPGREYFLAPDGGWYSIAAKFKDWEVVPTKVVLMYG SQVGNDQQAVNIANASLQDWSTYPAGTIALFHQRIATYSFTGNGAVIYTTNHGVVS VVRTTGGGWTMATGYT
part19	WEQVPMPTSKDAKGSRKTLEVVSQMTGVPIEYLMVFCALESNFDWTVKAGAGGS ATGWFQFINSTWDWVIGQHSAKYGLPADVGRRLRLDPRVNGLMGGEYIKYSMNVI KKGTGKDPTDIDLYLAHFLGPGTAVKWIKMPKTTIGSTAFPKEARANPSVFTDKRTG AQRTLGQIEQSFDERMAKYR
part21	GVKPLRRNVAIYGDSRTANCSSGSLPNKITENYGYASWLGQYSEGRIFFEPAFNFG VGGNTSAQWAARVGDVIASPSDVVVCLISTNDRTADFTLAQTQQNIEGVVMKLKQA GKIVVFINETPRGGANALTAPRQAIHEQVRAWINSYLPKLGVRVVDVWDKMTDTAA VTVDGLHFNPVGANIVGQALAREIQDLFNSPVPLPRVTSAYDSATNP
part24	MEYLSIWGDSRTAQNWNSAGNAATARGYAWWAEALSGRVRMHLKYNFGVSGDSI QQLLDRMNNDTANASGVKPSQVPPSHAVLHIGTNSINAENSVASCMYQLNQCIAWL QNKGHTVYVVSEWPRGQTVNAKEMSVLTPAAQKIMLGYAREVRKLARTKKVKVID VWPTMADPTRTDCRPRPNYLNNDSLHPSIGAGFLTGKLIAEALRENNAHKL
part25	NINAALNAVALKGGGKLIVRSHNGTDPIYLNGLVAIESNNVTLEFVSDVVYGSKGWM RISGGLAEIRRPGQIELLKLRLNSYANDDGYMVLPMRENNGSFLRVGDRITVRGEN DRNGKVLEKQVTIVKEIVGDDVICADEPDYTFKPTYPDSEYPEDLTTGTTISISVYSA MTGDTKNTDVIPVVDSTGFFAGDLVYVSDSRTERDMMQTVPPANLLSAANMEIVRI AAVEGNNLRLERAIRREYLTAWAGGVVKMDAIKNSHIKLRNVSWIGIQPDRKAHGA AINYGWACTVRVDDMQGRGGRKGIGVRVAYAYDCHVVDSKVYDAYSFLSAEGYGI SLYYSTMCSIRNCNASGNRHNYLLQTVTSCDVFDNNSNDDYISGIDLHGAGSVDCR VMRNRVSRSKSYADGVTNGGAIRNGNTAHTIGDHRTVIADNYIEGYLDVKCAAIDVS PASKDVIIRNNDIVDCAVGFRHYRINSSILPAQHTNRVTVEYNNFTRVAAPFDVNNYA NSIFDELIMIGNKSIENSTHFVVKNVAKVLAM
part26	DNINAALNDMQAKGGGKLVVRSHNGLDPIYINGLVAIENHNCTLEFVSDVVYGSKG WMRISGGLAEIRRPGQVELLKLRLNTYANDDGYMVLPMRENNGSFLRVGDRITVR GENDRNGKVLEKQVTIVKEIIGDDVICSDEPDYTFKPTYPDSDYPEDLTTGTTISISVY

Tile name	Amino acid sequence
	SAMTGNTKNTDVIPVVDSTGFFAGDLVYVSDSRTERDMMPTIPPANLLSAANMEIV RIAAVEGNNLRLERAIRREYLTEYAAGVVKMDAIKNSHIKLRNVTWNAPQPDRKAHG AAINYGWNCTIKVDDMYGRGGRKGIGIRIAYAYDCHAIDSKVYDAYSFLSAEGYGISL YYSTMCSVRNCIASGNRHNILLQTATSCDVFDNTCNDDYISGIDLHGAGSVDCRVM RNRLGRSKSYADGVTNGGAIRNGNTAHTIGDHRTVIADNYIEGYLDTKCAAIDVSPS SNDVIIRNNDIVDCQVGFRHYRVGSSINPAQHSNRVTVEYNNFTRVAQPFDVDNYA NSVFDELIMIGNK
part37	NNLTFNGVVDQRTNLWDALAPICKVGRAQIVRAGTRFQVSMVRKSKPVQMFSMGN IKKGTLAIDWLADDERANECHVSYYDKTDSGKQKTVIVPNLAARERGEEAKPTELTL YGVDNVAQATREGTLAMNMQQLLKTISFEAPISAIACTLGDVVAIQHDVPDWGQGG LTDVGST

Table S3. Summary frequency data for long read sequencing of total and active variant libraries.

Position	Part	Freq. lib. Input 1	Freq. lib. Input 2	Freq. lib input mean	Freq lib active	enrichment
Position 1	EAD50	13.63	12.84	13.235	14.18	7.14
Position 1	EAD51	12.71	13.5	13.105	16.17	23.39
Position 1	EAD53	14.24	12.45	13.345	1.24	-90.71
Position 1	Part14	14.24	14.02	14.13	11.69	-17.27
Position 1	Part19	14.4	14.68	14.54	19.65	35.14
Position 1	Part20	12.1	12.71	12.405	16.17	30.35
Position 1	Part28	6.58	9.04	7.81	7.21	-7.68
Position 1	Part29	12.1	10.75	11.425	13.68	19.74
Position 2	Link03	14.09	13.37	13.73	14.43	5.10
Position 2	Link04	14.24	13.76	14	13.93	-0.50
Position 2	Link05	10.26	11.8	11.03	7.46	-32.37
Position 2	Link06	15.77	14.02	14.895	22.14	48.64
Position 2	Link07	14.4	12.58	13.49	11.44	-15.20
Position 2	Link08	8.88	11.27	10.075	8.21	-18.51
Position 2	Link09	7.81	9.31	8.56	6.47	-24.42
Position 2	Part20	14.55	13.89	14.22	15.92	11.95
Position 3	Part01	8.12	8.26	8.19	3.23	-60.56
Position 3	Part02	8.58	7.99	8.285	11.44	38.08
Position 3	Part05	8.27	7.73	8	5.97	-25.38
Position 3	Part06	3.98	6.16	5.07	3.23	-36.29
Position 3	Part08	8.27	7.86	8.065	7.46	-7.50
Position 3	Part09	7.35	7.73	7.54	8.46	12.20
Position 3	Part12	8.27	7.73	8	8.96	12.00
Position 3	Part13	8.58	8.13	8.355	11.94	42.91
Position 3	Part18	0.46	2.1	1.28	3.48	171.88
Position 3	Part21	5.05	4.85	4.95	3.98	-19.60
Position 3	Part22	7.96	6.95	7.455	5.72	-23.27
Position 3	Part24	7.35	7.34	7.345	6.47	-11.91
Position 3	Part25	9.04	8.13	8.585	9.7	12.99
Position 3	Part26	8.58	8.26	8.42	9.95	18.17
Position 3	Part37	0.15	0.79	0.47	0	-100.00

Table S4. Summary of growth inhibition data for protein variants in Figure 1C-D.

Hit identifier	Parts	Ave. % growth inhibition (n = 8)	Highest % growth inhibition	Lowest % growth inhibition	P-value	Original screening % growth inhibition	Predicted size / kDa
**RLE B9**	Part14, linker3, EAD50	34	47	21	<0.0001	48	47.7
**RLE D9**	Part9, linker4, EAD50	17	31	-12.56	0.0742	33	46
**RLE E8**	Part25, linker3, EAD50	18	24	6	0.0457	30	54
**ELR C6**	Part28, linker9, Part25	28	57	-4	0.0006	44	89.8
**ELR F8**	Part19, linker9, Part25	16	51	5	0.0894	70	94.3
**ELR D5**	Part29, linker4, Part24	22	39	3	0.0056	42	56
**ELR D9**	Part19, linker9, Part5	37	57	6	<0.0001	47	31.8
**ELR E10**	Part29, linker5, Part24	36	50	17	<0.0001	46	54

Table S5. Strains used in this study.

Strain	Notes	Reference
Pseudomonas syringae pv. *actinidiae* ICMP 18884	*Psa* (WT)	[10]
Psa LPS attenuated strain A3	(*wbpl*::Tn)	[11]
Psa ICMP 24651 (Cu^R)	Resistant to 2 mM copper sulfate	[12]
Pseudomonas syringae pv. *syringae*	Co150	[13]
Pseudomonas syringae pv. *tomato* (*Pto*) DC3000		[14]
Pseudomonas fluorescens SBW25		[15]
Pseudomonas aeruginosa PAO1	Reference strain	[16]
Pectobacterium carotovorum RC5297	Carotovoricin-resistant	[17]
Staphylococcus aureus ATCC 25923	Reference strain	[18]
Escherichia coli DH5α	Thermofisher Scientific	Cat# 18265017
E. coli TOP10	Thermofisher Scientific	Cat# C404010
E. coli BL21(DE3)	Thermofisher Scientific	Cat# ECO114

Table S6. Oligonucleotides used in this study.

Primer name	Sequence	Description
PF4319	TGTGCTCTTCGAGAGGTCTCACCATGTTGACAGAAATTGACTACAAACAAGCCGCTG	EAD50_pos1_F
PF4320	TGTGCTCTTCGCTTGGTCTCAGCACCAATGAAACTTGCGTAGGCAGCAG	EAD50_pos1_R
PF4323	TGTGCTCTTCGAGAGGTCTCGCAGGCTTGACAGAAATTGACTACAAACAAGCCGCTG	EAD50_pos3_F
PF4325	TGTGCTCTTCGCTTGGTCTCATACTTAATGAAACTTGCGTAGGCAGCAG	EAD50_pos4_R
PF4354	TGTGCTCTTCGAGAGGTCTCACCATGTTGACAGAAATTGATTACAAACTAGCTGCAAG	EAD51_pos1_F
PF4355	TGTGCTCTTCGCTTGGTCTCAGCACCGAAACTTGCGTATGCAGCAGC	EAD51_pos1_R
PF4358	TGTGCTCTTCGAGAGGTCTCGCAGGCTTGACAGAAATTGATTACAAACTAGCTGCAAG	EAD51_pos3_F
PF4360	TGTGCTCTTCGCTTGGTCTCATACTTGAAACTTGCGTATGCAGCAGC	EAD51_pos4_R
PF8284	TGTGCTCTTCGAGAGGTCTCACCATGGAACTGAATAAAAAGGTCGTCG	EAD53_pos1_F
PF8285	TGTGCTCTTCGCTTGGTCTCAGCACCTTTGAAGGCACTCATGCAGTAG	EAD53_pos1_R
PF8286	TGTGCTCTTCGAGAGGTCTCGCAGGCGAACTGAATAAAAAGGTCGTCG	EAD53_pos3_F
PF8287	TGTGCTCTTCGCTTGGTCTCATACTTTTTGAAGGCACTCATGCAGTAG	EAD53_pos4_R
PF4378	TGTGCTCTTCGAGAGGTCTCACCATGCCAGGTATCACACGTGTTGGTATTG	part6_pos1_F
PF4379	TGTGCTCTTCGCTTGGTCTCAGCACCTGCGAAACAAGTATGCTTAGGGTC	part6_pos1_R
PF4380	TGTGCTCTTCGAGAGGTCTCGCAGGCCCAGGTATCACACGTGTTGGTATTG	part6_pos3_F
PF4382	TGTGCTCTTCGCTTGGTCTCATACTTTGCGAAACAAGTATGCTTAGGGTC	part6_pos4_R
PF4330	TGTGCTCTTCGAGAGGTCTCACCATGAACACATCTGGTAACGTGTCGGCTG	part8_pos1_F
PF4331	TGTGCTCTTCGCTTGGTCTCAGCACCGGAAGTTCTGATCCACAGTGTGTG	part8_pos1_R
PF4332	TGTGCTCTTCGAGAGGTCTCGCAGGCAACACATCTGGTAACGTGTCGGCTG	part8_pos3_F
PF4334	TGTGCTCTTCGCTTGGTCTCATACTTGGAAGTTCTGATCCACAGTGTGTG	part8_pos4_R
PF4335	TGTGCTCTTCGAGAGGTCTCACCATGACATTCTCTGTTCACGGTAGTGCAAC	part9_pos1_F
PF4336	TGTGCTCTTCGCTTGGTCTCAGCACCAATACCGATAGCCATGATATTGACTGTTG	part9_pos1_R
PF4337	TGTGCTCTTCGAGAGGTCTCGCAGGCACATTCTCTGTTCACGGTAGTGCAAC	part9_pos3_F
PF4338	TGTGCTCTTCGCTTGGTCTCATACTTAATACCGATAGCCATGATATTGACTGTTG	part9_pos4_R
PF4344	TGTGCTCTTCGAGAGGTCTCACCATGTCTGATCTGCCTAACGCACAAG	part12_pos1_F
PF4345	TGTGCTCTTCGCTTGGTCTCAGCACCAACGTACCCGATTGCTAGCCAAC	part12_pos1_R
PF4346	TGTGCTCTTCGAGAGGTCTCGCAGGCTCTGATCTGCCTAACGCACAAG	part12_pos3_F
PF4348	TGTGCTCTTCGCTTGGTCTCATACTTAACGTACCCGATTGCTAGCCAAC	part12_pos4_R

Primer name	Sequence	Description
PF4366	TGTGCTCTTCGAGAGGTCTCACCATGGCTACTACACCAAAGACGGTGC	part13_pos1_F
PF4367	TGTGCTCTTCGCTTGGTCTCAGCACCCTTCGAGGCCCACGCTTGG	part13_pos1_R
PF4368	TGTGCTCTTCGAGAGGTCTCGCAGGCGCTACTACACCAAAGACGGTGC	part13_pos3_F
PF4370	TGTGCTCTTCGCTTGGTCTCATACTTCTTCGAGGCCCACGCTTGG	part13_pos4_R
PF4388	TGTGCTCTTCGAGAGGTCTCACCATGAATCCGGTACTACATACACTCCCAC	part18_pos1_F
PF4389	TGTGCTCTTCGCTTGGTCTCAGCACCGGTGTACCCCGTGGCCATTG	part18_pos1_R
PF4390	TGTGCTCTTCGAGAGGTCTCGCAGGCAATCCGGTACTACATACACTCCCAC	part18_pos3_F
PF4392	TGTGCTCTTCGCTTGGTCTCATACTTGGTGTACCCCGTGGCCATTG	part18_pos4_R
PF4463	TGTGCTCTTCGAGAGGTCTCACCATGTGGGAACAAGTACCAATGCCTACATC	part19_pos1_F
PF4464	TGTGCTCTTCGCTTGGTCTCAGCACCACGATACTTAGCCATACGTTCATCAAAG	part19_pos1_R
PF4467	TGTGCTCTTCGAGAGGTCTCGCAGGCTGGGAACAAGTACCAATGCCTACATC	part19_pos3_F
PF4469	TGTGCTCTTCGCTTGGTCTCATACTTACGATACTTAGCCATACGTTCATCAAAG	part19_pos4_R
PF4393	TGTGCTCTTCGAGAGGTCTCACCATGGAGGTGGTTGCCCAGATGGTG	part20_pos1_F
PF4394	TGTGCTCTTCGCTTGGTCTCAGCACCTCCGTAAAGATACTAGGGTTAGCACG	part20_pos1_R
PF4397	TGTGCTCTTCGAGAGGTCTCGCAGGCGAGGTGGTTGCCCAGATGGTG	part20_pos3_F
PF4399	TGTGCTCTTCGCTTGGTCTCATACTTTCCGTAAAGATACTAGGGTTAGCACG	part20_pos4_R
PF4349	TGTGCTCTTCGAGAGGTCTCACCATGGGCGTCAAGCCGCTGCGTC	part21_pos1_F
PF4350	TGTGCTCTTCGCTTGGTCTCAGCACCCGGGTTGGTAGCAGAGTCATACG	part21_pos1_R
PF4351	TGTGCTCTTCGAGAGGTCTCGCAGGCGGCGTCAAGCCGCTGCGTC	part21_pos3_F
PF4353	TGTGCTCTTCGCTTGGTCTCATACTTCGGGTTGGTAGCAGAGTCATACG	part21_pos4_R
PF4438	TGTGCTCTTCGAGAGGTCTCACCATGGAATACTTGTCGATCTGGGGTGACAG	part24_pos1_F
PF4439	TGTGCTCTTCGCTTGGTCTCAGCACCGCTTGTGCGCGTTGTTCTCAC	part24_pos1_R
PF4440	TGTGCTCTTCGAGAGGTCTCGCAGGCGAATACTTGTCGATCTGGGGTGACAG	part24_pos3_F
PF4442	TGTGCTCTTCGCTTGGTCTCATACTTGCTTGTGCGCGTTGTTCTCAC	part24_pos4_R
PF4453	TGTGCTCTTCGAGAGGTCTCACCATGAACATCAACGCCGCGCTCAAC	part25_pos1_F
PF4454	TGTGCTCTTCGCTTGGTCTCAGCACCCATCGCCAGAACTTTGGCCAC	part25_pos1_R
PF4455	TGTGCTCTTCGAGAGGTCTCGCAGGCAACATCAACGCCGCGCTCAAC	part25_pos3_F
PF4457	TGTGCTCTTCGCTTGGTCTCATACTTCATCGCCAGAACTTTGGCCAC	part25_pos4_R
PF4443	TGTGCTCTTCGAGAGGTCTCACCATGGACAACATCAACGCCGCCCTG	part26_pos1_F

Primer name	Sequence	Description
PF4444	TGTGCTCTTCGCTTGGTCTCAGCACCCTTGTTGCCGATCAT GATCAGCTC	part26_pos1_R
PF4445	TGTGCTCTTCGAGAGGTCTCGCAGGCGACAACATCAACGC CGCCCTG	part26_pos3_F
PF4447	TGTGCTCTTCGCTTGGTCTCATACTTCTTGTTGCCGATCATG ATCAGCTC	part26_pos4_R
PF4470	TGTGCTCTTCGAGAGGTCTCACCATGGCCGCATTAAAGAAA GGCGATAC	part28_pos1_F
PF4471	TGTGCTCTTCGCTTGGTCTCAGCACCGCCTCGATGACTAGC AACCTTAG	part28_pos1_R
PF4474	TGTGCTCTTCTAGAGGTCTCGCAGGCGCCGCATTAAAGAAA GGCGATAC	part28_pos3_F
PF4400	TGTGCTCTTCGAGAGGTCTCACCATGGCTGCATTAAGAAAG GGTGATTCTGG	part29_pos1_F
PF4401	TGTGCTCTTCGCTTGGTCTCAGCACCCTTCCGGTGGGAGG CAACCTTG	part29_pos1_R
PF4404	TGTGCTCTTCGAGAGGTCTCGCAGGCGCTGCATTAAGAAA GGGTGATTCTGG	part29_pos3_F
PF4406	TGTGCTCTTCGCTTGGTCTCATACTTCTTCCGGTGGGAGGC AACCTTG	part29_pos4_R
PF4476	TGTGCTCTTCGCTTGGTCTCATACTTGCCTCGATGACTAGC AACCTTAG	part28_pos4_R
PF4423	TGTGCTCTTCGAGAGGTCTCACCATGAACAATCTGACCTTC AACGGCGTAG	part37_pos1_F
PF4424	TGTGCTCTTCGCTTGGTCTCAGCACCGGTGCTACCCACATC GGTG	part37_pos1_R
PF4425	TGTGCTCTTCGAGAGGTCTCGCAGGCAACAATCTGACCTTC AACGGCGTAG	part37_pos3_F
PF4426	TGTGCTCTTCGAGAGGTCTCGGAAGCAACAATCTGACCTTC AACGGCGTAG	part37_pos4_F
PF4427	TGTGCTCTTCGCTTGGTCTCATACTTGGTGCTACCCACATC GGTG	part37_pos4_R
PF4632	TAATACGACTCACTATAGGGGAATTGTGAG	F screening primer pVTD3
PF4633	TTATTGCTCAGCGGTGGCAGC	R screening primer pVTD3
PF4714	CTCACATGTTCTTTCCTGCG	F screening primer PVTE
PF4715	TCTCATGAGCGGATACATATTTG	R screening primer PVTE
PF6711	TGTGCTCTTCGCTTGGTCTCATACTTCAGCTTGTGCGCGTT GTTCTCAC	Part24 pos 4 reverse primer
PF6712	CGTCGATCGCATCGGCTTTCGATTACACG	Forward part29 E112A
PF6713	GAAAGCCGATGCGATCGACGCAAACGTG	Reverse part29 E112A
PF6714	CTGAGCTCTTCGCAGCTCACTTCTTTGG	Forward part29 Y194F
PF6715	GTGAGCTGCGAAGAGCTCAGTATCGGTAGC	Reverse part29 Y194F

References

(1) Frampton, R. A.; Taylor, C.; Holguín Moreno, A. V.; Visnovsky, S. B.; Petty, N. K.; Pitman, A. R.; Fineran, P. C. Identification of Bacteriophages for Biocontrol of the Kiwifruit Canker Phytopathogen *Pseudomonas Syringae* Pv. *Actinidiae*. *Appl. Environ. Microbiol.* **2014**, *80* (7), 2216–2228. https://doi.org/10.1128/AEM.00062-14.

(2) Wojtus, J. K.; Frampton, R. A.; Warring, S.; Hendrickson, H.; Fineran, P. C. Genome Sequence of a Jumbo Bacteriophage That Infects the Kiwifruit Phytopathogen *Pseudomonas Syringae* Pv. *Actinidiae*. *Microbiol. Resour. Announc.* **2019**, *8* (22). https://doi.org/10.1128/MRA.00224-19.

(3) Latz, S.; Krüttgen, A.; Häfner, H.; Buhl, E.; Ritter, K.; Horz, H.-P. Differential Effect of Newly Isolated Phages Belonging to PB1-Like, PhiKZ-Like and LUZ24-Like Viruses against Multi-Drug Resistant *Pseudomonas Aeruginosa* under Varying Growth Conditions. *Viruses* **2017**, *9* (11), 315. https://doi.org/10.3390/v9110315.

(4) Mesyanzhinov, V. V.; Robben, J.; Grymonprez, B.; Kostyuchenko, V. A.; Bourkaltseva, M. V.; Sykilinda, N. N.; Krylov, V. N.; Volckaert, G. The Genome of Bacteriophage ΦKZ of Pseudomonas Aeruginosa. *J. Mol. Biol.* **2002**, *317* (1), 1–19. https://doi.org/10.1006/jmbi.2001.5396.

(5) Briers, Y.; Volckaert, G.; Cornelissen, A.; Lagaert, S.; Michiels, C. W.; Hertveldt, K.; Lavigne, R. Muralytic Activity and Modular Structure of the Endolysins of *Pseudomonas Aeruginosa* Bacteriophages ΦKZ and EL. *Mol. Microbiol.* **2007**, *65* (5), 1334–1344. https://doi.org/10.1111/j.1365-2958.2007.05870.x.

(6) Chertkov, O. V; Armeev, G. A.; Uporov, I. V; Legotsky, S. A.; Sykilinda, N. N.; Shaytan, A. K.; Klyachko, N. L.; Miroshnikov, K. A. Dual Active Site in the Endolytic Transglycosylase Gp144 of Bacteriophage PhiKZ. *Acta Naturae* **2017**, *9* (1), 81–87.

(7) Bajaj, K.; Madhusudhan, M. S.; Adkar, B. V; Chakrabarti, P.; Ramakrishnan, C.; Sali, A.; Varadarajan, R. Stereochemical Criteria for Prediction of the Effects of Proline Mutations on Protein Stability. *PLoS Comput. Biol.* **2007**, *3* (12), e241. https://doi.org/10.1371/journal.pcbi.0030241.

(8) Zhang, H.; Wu, H.; Xia, H.; Zhong, C.; Li, L.; Zeng, C. Genomic Characterization of Two Nickie-like Bacteriophages That Infect the Kiwifruit Canker Phytopathogen *Pseudomonas Syringae* Pv. *Actinidiae*. *Arch. Virol.* **2022**, *167* (8), 1713–1715. https://doi.org/10.1007/s00705-022-05440-8.

(9) Briers, Y.; Lavigne, R.; Volckaert, G.; Hertveldt, K. A Standardized Approach for Accurate Quantification of Murein Hydrolase Activity in High-Throughput Assays. *J. Biochem. Biophys. Methods* **2007**, *70* (3), 531–533. https://doi.org/10.1016/j.jbbm.2006.10.009.

(10) Templeton, M. D.; Warren, B. A.; Andersen, M. T.; Rikkerink, E. H. A.; Fineran, P. C. Complete DNA Sequence of *Pseudomonas Syringae* Pv. *Actinidiae*, the Causal Agent of Kiwifruit Canker Disease. *Genome Announc.* **2015**, *3* (5), e01054-15. https://doi.org/10.1128/genomeA.01054-15.

(11) Mesarich, C. H.; Rees-George, J.; Gardner, P. P.; Ghomi, F. A.; Gerth, M. L.; Andersen, M. T.; Rikkerink, E. H. A.; Fineran, P. C.; Templeton, M. D. Transposon Insertion Libraries for the Characterization of Mutants from the Kiwifruit Pathogen *Pseudomonas Syringae* Pv. *Actinidiae*. *PLoS One* **2017**, *12* (3), 1–21. https://doi.org/10.1371/journal.pone.0172790.

(12) *Manaaki Whenua Landcare Research NZ*. Pseudomonas syringae pv. actinidiae ICMP 24651 – Systematics Collection Data.

(13) Marroni, M. V.; Casonato, S.; Visnovsky, S. B.; Pitman, A. R.; Beresford, R. M.; Jones, E. E. Genetic Characterization and Prevalence of *Pseudomonas Syringae* Strains from Sweet Cherry Orchards in New Zealand. *Plant Pathol.* **2023**, *72* (9), 1673–1686. https://doi.org/10.1111/ppa.13775.

(14) Buell, C. R.; Joardar, V.; Lindeberg, M.; Selengut, J.; Paulsen, I. T.; Gwinn, M. L.; Dodson, R. J.; Deboy, R. T.; Durkin, A. S.; Kolonay, J. F.; Madupu, R.; Daugherty, S.; Brinkac, L.; Beanan, M. J.; Haft, D. H.; Nelson, W. C.; Davidsen, T.; Zafar, N.; Zhou, L.; Liu, J.; Yuan, Q.; Khouri, H.; Fedorova, N.; Tran, B.; Russell, D.; Berry, K.; Utterback, T.; Van Aken, S. E.; Feldblyum, T. V.; D'Ascenzo, M.; Deng, W.-L.; Ramos, A. R.; Alfano, J. R.; Cartinhour, S.; Chatterjee, A. K.; Delaney, T. P.; Lazarowitz, S. G.; Martin, G. B.; Schneider, D. J.; Tang, X.; Bender, C. L.; White, O.; Fraser, C. M.; Collmer, A. The Complete Genome Sequence of the Arabidopsis and Tomato Pathogen *Pseudomonas Syringae* Pv. *Tomato* DC3000. *Proc. Natl. Acad. Sci.* **2003**, *100* (18), 10181–10186. https://doi.org/10.1073/pnas.1731982100.

(15) Silby, M. W.; Cerdeño-Tárraga, A. M.; Vernikos, G. S.; Giddens, S. R.; Jackson, R. W.; Preston, G. M.; Zhang, X.-X.; Moon, C. D.; Gehrig, S. M.; Godfrey, S. A.; Knight, C. G.;

Malone, J. G.; Robinson, Z.; Spiers, A. J.; Harris, S.; Challis, G. L.; Yaxley, A. M.; Harris, D.; Seeger, K.; Murphy, L.; Rutter, S.; Squares, R.; Quail, M. A.; Saunders, E.; Mavromatis, K.; Brettin, T. S.; Bentley, S. D.; Hothersall, J.; Stephens, E.; Thomas, C. M.; Parkhill, J.; Levy, S. B.; Rainey, P. B.; Thomson, N. R. Genomic and Genetic Analyses of Diversity and Plant Interactions of *Pseudomonas Fluorescens*. *Genome Biol.* **2009**, *10* (5), R51. https://doi.org/10.1186/gb-2009-10-5-r51.

(16) Stover, C. K.; Pham, X. Q.; Erwin, A. L.; Mizoguchi, S. D.; Warrener, P.; Hickey, M. J.; Brinkman, F. S. L.; Hufnagle, W. O.; Kowalik, D. J.; Lagrou, M.; Garber, R. L.; Goltry, L.; Tolentino, E.; Westbrock-Wadman, S.; Yuan, Y.; Brody, L. L.; Coulter, S. N.; Folger, K. R.; Kas, A.; Larbig, K.; Lim, R.; Smith, K.; Spencer, D.; Wong, G. K.-S.; Wu, Z.; Paulsen, I. T.; Reizer, J.; Saier, M. H.; Hancock, R. E. W.; Lory, S.; Olson, M. V. Complete Genome Sequence of *Pseudomonas Aeruginosa* PAO1, an Opportunistic Pathogen. *Nature* **2000**, *406* (6799), 959–964. https://doi.org/10.1038/35023079.

(17) Birkholz, N.; Jackson, S.; Fagerlund, R. D.; Fineran, P. C. A Mobile Restriction–Modification System Provides Phage Defence and Resolves an Epigenetic Conflict with an Antagonistic Endonuclease. *Nucleic Acids Res.* **2022**, *In press*.

(18) Treangen, T. J.; Maybank, R. A.; Enke, S.; Friss, M. B.; Diviak, L. F.; Karaolis, D. K. R.; Koren, S.; Ondov, B.; Phillippy, A. M.; Bergman, N. H.; Rosovitz, M. J. Complete Genome Sequence of the Quality Control Strain *Staphylococcus Aureus* Subsp. *Aureus* ATCC 25923. *Genome Announc.* **2014**, *2* (6). https://doi.org/10.1128/genomeA.01110-14.

(19) Warring, S. L.; Malone, L. M.; Jayaraman, J.; Easingwood, R. A.; Rigano, L. A.; Frampton, R. A.; Visnovsky, S. B.; Addison, S. M.; Hernandez, L.; Pitman, A. R.; Lopez Acedo, E.; Kleffmann, T.; Templeton, M. D.; Bostina, M.; Fineran, P. C. A Lipopolysaccharide-dependent Phage Infects a *Pseudomonad* Phytopathogen and Can Evolve to Evade Phage Resistance. *Environ. Microbiol.* **2022**, *24* (10), 4834–4852. https://doi.org/10.1111/1462-2920.16106.

(20) Frampton, R.; Acedo, E.; Young, V.; Chen, D.; Tong, B.; Taylor, C.; Easingwood, R.; Pitman, A.; Kleffmann, T.; Bostina, M.; Fineran, P. Genome, Proteome and Structure of a T7-Like Bacteriophage of the Kiwifruit Canker Phytopathogen *Pseudomonas Syringae* Pv. *Actinidiae*. *Viruses* **2015**, *7* (7), 3361–3379. https://doi.org/10.3390/v7072776.

(21) Wojtus, J. K.; Fitch, J. L.; Christian, E.; Dalefield, T.; Lawes, J. K.; Kumar, K.; Peebles, C. L.; Altermann, E.; Hendrickson, H. L. Complete Genome Sequences of Three Novel *Pseudomonas Fluorescens* SBW25 Bacteriophages, Noxifer, Phabio, and Skulduggery. *Genome Announc.* **2017**, *5* (31), e00725-17. https://doi.org/10.1128/genomeA.00725-17.
